# Inferring the Structure of Social Contacts from Demographic Data in the Analysis of Infectious Diseases Spread

**DOI:** 10.1371/journal.pcbi.1002673

**Published:** 2012-09-13

**Authors:** Laura Fumanelli, Marco Ajelli, Piero Manfredi, Alessandro Vespignani, Stefano Merler

**Affiliations:** 1 Bruno Kessler Foundation, Trento, Italy; 2 Department of Statistics and Mathematics Applied to Economics, University of Pisa, Pisa, Italy; 3 Department of Health Sciences and College of Computer and Information Sciences, Northeastern University, Boston, Massachusetts, United States of America; 4 Institute for Quantitative Social Sciences at Harvard University, Cambridge, Massachusetts, United States of America; 5 Institute for Scientific Interchange Foundation, Turin, Italy; Pennsylvania State University, United States of America

## Abstract

Social contact patterns among individuals encode the transmission route of infectious diseases and are a key ingredient in the realistic characterization and modeling of epidemics. Unfortunately, the gathering of high quality experimental data on contact patterns in human populations is a very difficult task even at the coarse level of mixing patterns among age groups. Here we propose an alternative route to the estimation of mixing patterns that relies on the construction of virtual populations parametrized with highly detailed census and demographic data. We present the modeling of the population of 26 European countries and the generation of the corresponding synthetic contact matrices among the population age groups. The method is validated by a detailed comparison with the matrices obtained in six European countries by the most extensive survey study on mixing patterns. The methodology presented here allows a large scale comparison of mixing patterns in Europe, highlighting general common features as well as country-specific differences. We find clear relations between epidemiologically relevant quantities (reproduction number and attack rate) and socio-demographic characteristics of the populations, such as the average age of the population and the duration of primary school cycle. This study provides a numerical approach for the generation of human mixing patterns that can be used to improve the accuracy of mathematical models in the absence of specific experimental data.

## Author Summary

The dynamics of infectious diseases caused by pathogens transmissible from human to human strongly depends on contact patterns between individuals. High quality observational data on contact patterns, usually presented in the form of age-specific contact matrices, are difficult to gather and are currently available only for few countries worldwide. Here we propose a computational approach, based on the simulation of a virtual society of agents, allowing the estimation of contact patterns by age for 26 European countries. We validate the estimated contact matrices against those obtained by the most extensive field study on contact patterns, with data collected in eight European countries. We show that our contact matrices share some common features, e.g. individuals tend to mix preferentially with individuals their own age, and country-specific differences, which can be partly explained by differences in population structures due to different demographic trajectories followed after WWII. Our analysis highlights well defined correlations between epidemiological parameters and socio-demographic features of the populations. This study provides the first estimates of contact matrices for many European countries where specific experimental data are still not available.

## Introduction

The accurate characterization of the structure of social contacts in mathematical and computational models of infectious disease transmission is a key element in the assessment of the impact of epidemic outbreaks and in the evaluation of effective control measures. For instance, the transmissibility potential of a disease and the final epidemic size strongly depend on mixing patterns between individuals of the population, which in turn depend on socio-demographic parameters (e.g. household size, fraction of workers and students in the population) [1]–[5]. For this reason, several efforts have been recently carried out in order to obtain contact data with the aim of quantifying “who meets whom (where, when, how long and how often)” [6]–[12], possibly also over time [13], [14]. Empirical data collection on a large scale is however extremely difficult and although several models tackling both new emerging epidemics and endemic diseases have introduced a significant amount of information on contact patterns [3], [5], [15]–[31], it is clear that the increasing use of data-driven models in the support of public health decisions is calling for novel approaches to the estimation of mixing patterns in human populations.

In this study we propose to overcome the above challenges by developing a general computational approach to derive mixing patterns from routinely collected socio-demographic data. In particular we focus on contact matrices by age of 26 European countries for which we are in the position to construct a synthetic society in the computer by integrating available social and census data. The use of contact matrices is the simplest way to improve on the homogeneous mixing assumption while at the same time preserving the analytical transparency of the model. The proposed approach is based on the simulation of a virtual society of agents that allows the estimate of contact matrices by age in different social settings: household, school, workplace and general community. Unlike classical agent based approaches of epidemic transmission [3], [5], [15], [17], [19], [32] and network models [33], [34], which are aimed at characterizing the spatio-temporal spread of epidemics tagging each individual in the population with a set of social attributes, we use the same detailed information on social contacts to construct contact matrices by age in the different settings to be used in compartmental models. This approach integrates population details, providing an effective description of the population structure to be used in computational models relying on compartmental schemes both at the continuous and individual based scale. Such a strategy might be very convenient to reduce the computational time demand in the analysis of large scale geographical models [21], [35]–[39].

Those matrices are appropriately combined in order to obtain the overall “adequate” total contact matrix for influenza-like-illness. In order to validate the proposed approach we compare the obtained contact matrices by age with the results of the Polymod study [9], the first large-scale survey on social mixing patterns relevant to infectious disease transmission. We show that the synthetically generated matrices share several common features with the Polymod matrices, e.g. strong assortativeness and the presence of similar secondary diagonal contact patterns. We integrate the synthetic contact matrices in a simple model for acute infectious diseases and highlight the role played by social and demographic factors in determining the different epidemic patterns in different countries. Further analysis and validation on the derived contact matrices is provided by investigating seroprevalence data for the 2009 H1N1 influenza pandemic in the UK.

The proposed method is extremely general and can be readily exported to other countries in the world for which the necessary social and demographic data can be gathered. We consider this approach an important step in order to overcome the current difficulties in real data gathering. Furthermore the computational path to the estimate of contact matrices represents a convenient scheme for the introduction of detailed individual based information in a wide range of modeling approaches working at the population level. For this reason we publicly release the entire collection of contact matrices to the scientific community (see Table S1 and S2; public download is also available at http://www.epiwork.eu/resources/matrices).

## Materials and Methods

### Socio-demographic data

In order to provide a quantitative estimate of contact matrices for 26 European countries we used highly detailed data on the country-specific socio-demographic structures (e.g., household size and composition, age structure, rates of school attendance, etc.) available at the Statistical Office of the European Commission [40]. These data were used to generate highly detailed synthetic populations for all countries of the study area. More specifically, census data on frequencies of household size and type, age of household components by size were used to group individuals into households. Data on rates of employment/inactivity and school attendance by age, structure of educational systems, school and workplace size allowed us to either assign individuals to schools and workplaces or tag them as inactive, according to their age.

The procedure to generate the synthetic populations is quite standard in the context of individual based models and is therefore discussed in detail in Text S1. In the following paragraph we present the approach used in the computation of the synthetic contact matrices.

### Computing contact matrices

The mathematical representation of epidemics relies on the description of the transmission process which is usually modeled through the force of infection, that is the rate at which a susceptible individual acquires the infection because of the interactions with infectious individuals. This quantity is proportional to the number of infectious individuals, the specific transmission probability of the infection during a contact and the overall rate of contacts of each individual with other individuals in the population. Although a vast majority of studies assumes the population as homogeneous –all individuals are equal with same average contact rate– the social and demographic structure of the population is generally reflected in heterogeneous contact patterns among individuals. Age is obviously one of the main determinants of the mixing pattern of individuals. Children tend to spend more time with children and members of their household, active adults mix with individuals in their workplace etc. Mixing patterns by age are generally defined by a contact matrix whose elements 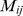 represent the average frequency of “adequate” contacts that an individual of age 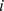 has with individuals aged 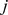. We define a contact as “having shared the same physical environment” [11] (e.g., the same household, or the same school or workplace). To compute age-specific contacts we postulate that at the finest scale of the single units, e.g. single households or schools, mixing is homogeneous. This hypothesis is necessary given the lack of information on contacts at this fine scale. By aggregating units we then compute “setting-specific” contact matrices, represented by the four matrices accounting for contacts within household members (matrix 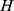), within schoolmates/teachers (matrix 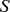), within workplace colleagues (matrix 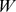) and in the general community (matrix 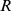). Finally, the overall age-specific matrix 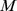 is computed as a linear combination of the four matrices 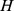, 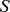, 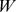, 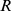.

**Figure 1:**
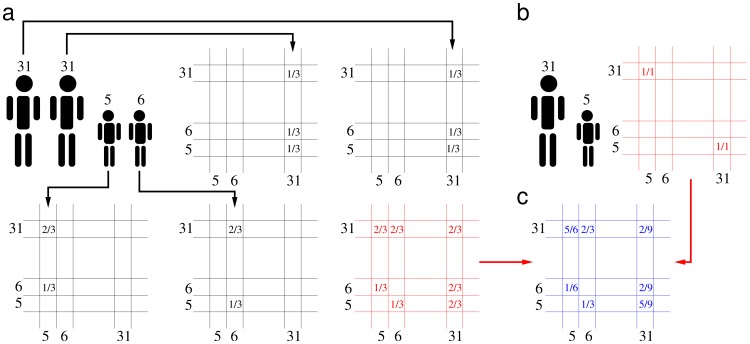
Example of the computation of household contact matrix. **a** Computation of contact frequencies for every member of a household composed by two adults aged 31 and two children of 5 and 6 years old. The sum of the four contributions gives contact frequencies within this household (in red). **b** Contact frequencies within a household composed of an adult aged 31 and a child aged 5. **c** Assuming that these two households constitute the whole population, the frequency of household contacts that individuals of age 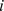 have with individuals aged 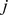 is given by the sum of the contributions from each household, divided by the number of individuals aged 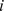 having at least one household contact.

To give an example, let us see in more detail the computation of the matrix of contacts within households, 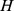. For each individual 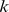 of age 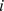, living in household 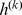 of size 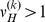, the household contacts with individuals of age 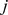 are defined as the set of individuals of age 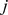 living in household 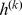. We denote the number of individuals in this set by 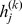. Then, once 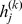 is computed, we obtain the probability that individual 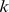 has contacts with individuals of age 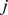 by dividing the number of contacts with members of age 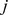 by 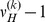, which represents the number of individuals living in the same household as 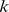. The expression for the frequency of contacts within households 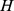 between individuals of ages 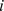 and 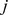 is then
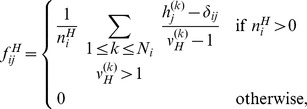
where 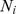 is the total number of individuals of age 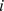 in the population, 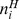 is the number of individuals of age 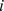 with at least one contact in the household (note that some individuals may have zero contacts) and 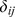 is the Kronecker delta function that allows excluding individual 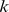 from the set of her/his own contacts. A straightforward example of the computation of household contacts frequencies is provided in Figure 1.

In order to transform the frequency of contacts into contact matrices relevant for infectious disease spreading, we need to consider the following quantities:

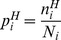
 which is the probability for age bracket 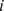 to have at least one contact in the household. This probability is less than one as individuals may live alone;

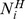
 which is the rate of total effective contacts in the household for individuals of age 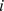. This number depends on the time scale, the type of disease etc.

We can therefore define the synthetic contact matrix for households
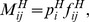
which provides the relative frequency of contact among age classes. In order to obtain the rate of effective contacts between classes 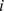 and 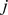 we need then to consider the product 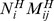. In the following, in the lack of a better knowledge, we will assume that the effective contact rate is age independent: 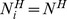. Expressions analogous to the previous one can be used to compute the frequency of contacts by age within schools (superscript 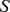) and within workplaces (superscript 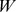); the matrix for schools 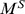 is given by the sum of the matrices for each school level, from pre-primary to higher education. As regards the frequency of contacts in the general community, we assume homogeneous mixing among individuals, thus the columns of the matrix (superscript 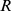) are proportional to the number of individuals by age.

In order to define the “adequate” contact matrix [41] we assume that the total rate of effective contacts in the households can be expressed as 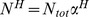, where 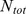 is the total rate of effective contacts and 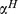 is a constant providing the fraction of effective contacts within the household. Similar expressions can be written for all other settings. Since there is evidence that infection transmission is not uniform by setting [42], [43] and the relevance of every setting (household, school, work, general community) in the transmission depends on the pathogen responsible for the disease, in principle it is possible to estimate empirically the fraction of transmission events 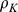 in each setting 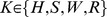. In this study, in order to give a baseline, we assume values for influenza-like-illness (ILI) transmission following empirical estimates of the proportions of transmission in the different settings [5], [42], [44]–[46]: 0.3 in households, 0.18 in schools, 0.19 in workplaces and 0.33 in the general community. Although these weights are related to influenza, they have already been used in [11] to generate a synthetic contact matrix for the Italian population, which has been shown to capture Varicella and Parvovirus B19 data.

For each setting 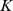 we obtain the condition
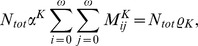
where 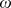 is the maximum age of the population. This set of equations readily provides the values
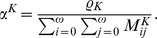
The resulting adequate contact matrix 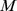 is therefore defined as the linear combination of the contact matrices in each setting:
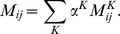
This notion of adequate matrix of total contacts appears to be appropriate, up to a scale factor, when the transmission coefficients of the infection depend only on settings (i.e. they are age-independent), and provided that the proportions of transmission in the different settings are roughly constant during the course of the epidemic.

Matrix 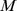 defines the contact pattern among ages up to a constant 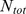 that has to be considered as an appropriate rescaling factor when comparing matrices defined according to different time scales or data aggregation processes. In the study of epidemic processes, by assuming that the probability of transmission 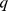 per effective contact is constant, the contact rate 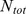 is usually absorbed in the definition of the transmissibility rate 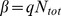 that is used as the scaling factor determining the reproduction number 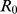 that characterizes the specific pathogen transmission. 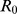 essentially represents the average number of secondary cases generated by a primary case in a completely susceptible population [47], and it is therefore the threshold parameter determining the dynamics of the epidemic.

Remarkably, our matrices are computed by considering one-year age brackets, from 0 to 100 and over years; this is the most refined version of our data on frequencies of contacts. They can however be aggregated in different ways, depending on the purpose for which they are used: for instance, for childhood diseases one may prefer to group contact data for children according to educational levels.

Although we are dealing with very detailed data on the socio-demographic structures of European countries, there are a number of limitations and assumptions that it is worth stating. First of all, although the relevant statistics could be gathered from other sources, we consider Eurostat as the only source of data on occupation rates. This is the reason why we decided to exclude Belgium, Poland and Malta from our study in view of the incomplete information on employment and schooling rates. Furthermore, the different household structures considered in our virtual society cover about the 95% of the total number of households in Europe. We do not allow however families with an aggregate member or non-private households (such as rest homes, dorms, religious and military institutions). Finally, another limitation lies in the assumption of homogeneous mixing for the contacts occurring in the community at large (i.e., not occurring between household members, schoolmates and work colleagues). In fact, this implies to disregard any kind of preferential mixing, e.g. by age, and the level of activity of individuals, which may vary by age, as documented by Polymod data [9]. Clearly, the availability of more precise and complete data on any aspect of the socio-demographic structure of a population (e.g., number and composition of non-private households; size and attendance of nurseries) would allow a refinement of our virtual society.

### SIR model with heterogeneous mixing patterns

In the classic SIR model the population is divided into three compartments: susceptible (individuals that can acquire infection), infectious (individuals that have been infected and are able to transmit the pathogen) and recovered (individuals that are immune to the disease–e.g. because they recovered from the infection). In order to include the mixing patterns encoded in the contact matrices, each group is characterized by an age structure. Every susceptible individual of age 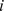 (belonging to the 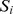 group) experiences an age-specific force of infection 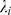, which is determined by the average frequency 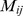 of “adequate” contacts that an individual of age 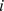 has with individuals aged 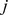, by the probability of contacting infectious individuals from every age class 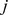, and by a transmissibility 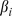 that accounts for the probability of infection per contact. The force of infection yields the rate of transition of susceptible individuals into the infectious state 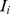; individuals then leave this status according to the recovery rate 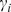 (the inverse of the duration of the infectious period), entering the recovered compartment 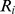. For the sake of simplicity we consider age independent transmissibility 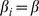 and recovery rate 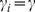. The set of equations governing the SIR model can be thus written as
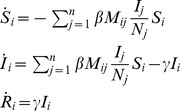
(1)where 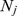 is the number of individuals in the population of age 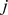 and 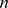 is the number of age classes considered. 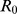 is calculated as the spectral radius of the next generation matrix [48], that is 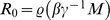.

To analyze post-pandemic H1N1 serological data collected in England and Wales in fall 2009 [49], we make use of a slightly modified version of model (1) accounting for age-specific susceptibility to infection, which is acknowledged as a further critical determinant of the force of infection for influenza. In particular, the equations for susceptibles and infectious become:
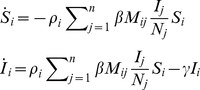
where 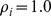 if 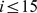, 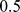 otherwise as resulting from estimates reported in [50], [51].

## Results/Discussion

### Synthetic contact matrices by age

**Figure 2:**
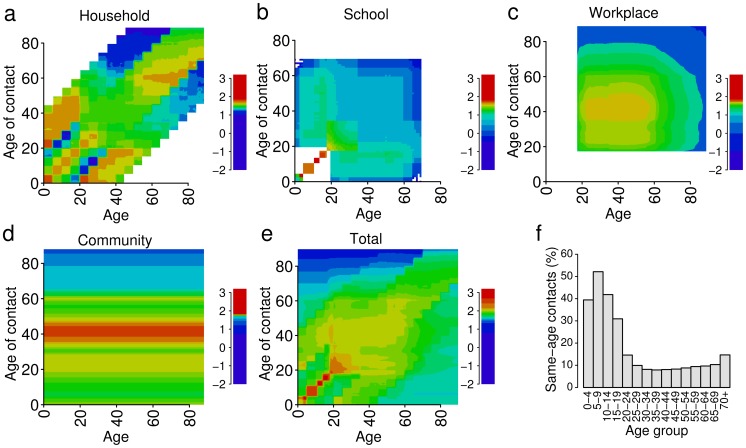
Mixing patterns by age in the UK. Representations in logarithmic scale of contact matrices by one-year age brackets for the United Kingdom in the different social settings. Frequency of contacts (in arbitrary units) increases from blue to red. **a** Household. **b** School. **c** Workplace. **d** General community. **e** The total matrix obtained as a linear combination of the matrices represented in **a–d**; the coefficients used are the proportions of transmission in the four settings: 0.3 in households, 0.18 in schools, 0.19 in workplaces and 0.33 in the general community [3], [11], [42], [44]–[46]. **f** Proportions of contacts with individuals of the same age group, from the total matrix.

Figure 2 shows the contact matrices obtained for the United Kingdom. They present a number of clear features that reflect the socio-demographic structure of the population: i) the matrix for households (Figure 2a) shows a dominant diagonal, representing contacts with siblings for young individuals, and with spouse for adults, whose ages are generally similar. There are also a lower and an upper diagonal, accounting for contacts that parents have with their children and vice versa; these contacts are generally absent for people aged over 60; ii) the structure of the educational system is clear from the matrix of contacts at school (Figure 2b) as young individuals mix mostly with persons of similar age, belonging to same school level; iii) in the workplaces (Figure 2c) most contacts occur between people from 20 to 60 years old, corresponding to the working age population. Finally, the matrix for contacts in the community, obtained by assuming homogeneous mixing, reflects the age structure of the overall population only (Figure 2d).

In Figure 2e the “adequate” contact matrix is reported; for young individuals, contacts within schoolmates of similar age are represented on the main diagonal; mixing in workplaces is prevalent for adults aged 20 to 60 years; people aged more than 65 years have most contacts with people of similar age. The difference between the upper left and the lower right entries of the matrix is a consequence of the age structure of the population, which is characterized by a small fraction of individuals aged over 80. Figure 2f reports the proportions of same-age contacts by 5-year age groups of this matrix: from 40 to more than 50% of the contacts of individuals under 15 years are within the same age group, basically with schoolmates and siblings at home. Adults contacts instead are much less assortative because the working period spans a wide range of ages.

### Country-specific features of synthetic contact matrices

Although similar attributes can be observed in the synthetic contact matrices for all 26 countries under consideration (the representation of all matrices other than UK are reported in Text S1), some features are distinctive of specific regions. For instance, household mixing is always characterized by the three diagonals representing contacts between siblings/spouse or parents and children, but in Northern Europe and France the upper diagonal is shorter, reflecting the fact that people tend to leave home earlier than in the other countries. The contribution of educational systems is always represented on the lower part of the main diagonal: the observed heterogeneity among countries is driven by the organization of school cycles (e.g. the arrangement of primary and lower secondary schools into either single or separate structures is clearly visible). The central part of the matrices is associated to contacts at work, which depend on the age structure of the working population. Moreover, we can observe that mixing with individuals of about 60 years of age tends to be higher in Northern countries and lower in the others, particularly in Southern Europe: this is probably due to differences in retirement age [52].

In order to infer more rigorously whether similarities between contact matrices can be identified to characterize specific groups of countries, we use a hierarchical cluster algorithm. The algorithm uses the average dissimilarity between two matrices 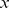 and 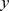 (treated as vectors) as measured by the Canberra distance 
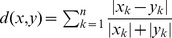
; this choice was made because the entries of contact matrices range over several orders of magnitude, and this distance, differently from 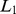 and 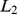 distances, is appropriate to measure average relative dissimilarities rather than absolute dissimilarities [53]. We found that contact matrices can be clustered in a way mainly reflecting the geographical location of the country. This may be motivated with the observation that neighboring states, for historical and cultural reasons, show marked similarities in school organization and demographic structures. The latter are well explained by the common demographic trajectories followed after World War II with respect to major structural changes, from the baby boom in the Sixties, to the fall towards low fertility [54].

**Figure 3:**
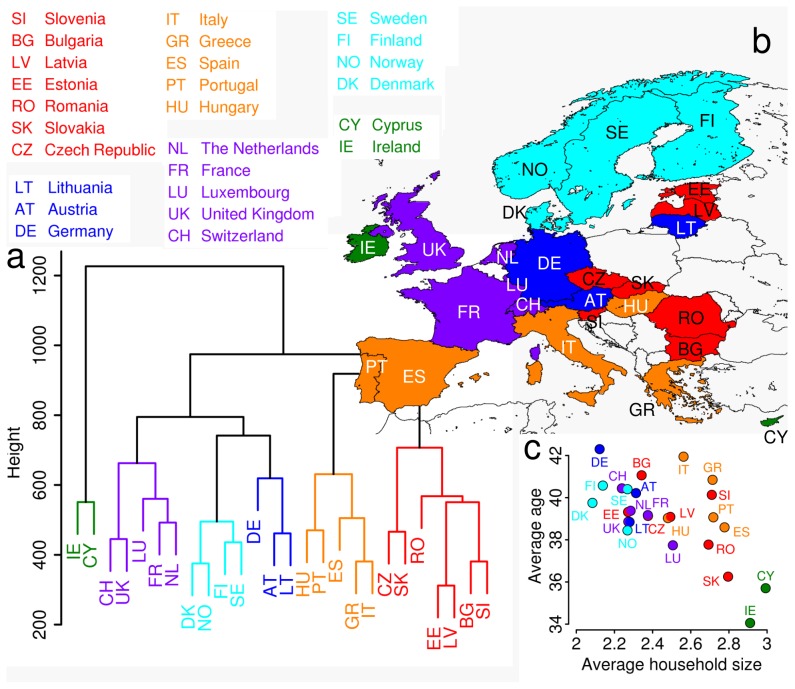
Characterization of synthetic contact matrices. Clustering of countries on the basis of total matrices. **a** Dendrogram of cluster analysis based on the Canberra distance. **b** Map of Europe and grouping of countries made by the algorithm; countries having the same color belong to the same cluster. **c** Average age and household size for the 26 countries considered. Colors as in the map.

In particular, we isolated four main clusters (see Figure 3): Ireland and Cyprus, Eastern, Southern and Northern Europe. This grouping can be partly explained in terms of some macroscopic indicators such as average age (which is a proxy for the number of students in the population), household size (see inset of Figure 3) and school organization. For instance, Ireland and Cyprus are the youngest European countries: the average ages are 34.1 and 35.7 years respectively, while the overall average age in Europe is 39.2. Moreover, they have the largest household size (2.88 individuals for Ireland, 2.98 for Cyprus, 2.49 for Europe). All Scandinavian countries (Norway, Sweden, Finland, Denmark) are grouped into a unique sub-cluster which is characterized by large average age and small household size. Eastern and Southern Europe are similar in terms of average age and household size, but in Southern countries elementary and lower secondary schools are organized as separate structures, whereas a unique cycle is predominant in Eastern countries. Interestingly, in Eastern Europe two sub-clusters can be identified: Czech Republic-Slovakia, which became two independent states few years ago, and Estonia-Latvia. Lithuania instead is grouped with Germany and Austria: this distinction may be due, at least partially, to Lithuanian school organization, which is similar to Central and Western Europe.

### Validation with Polymod contact matrices

**Figure 4:**
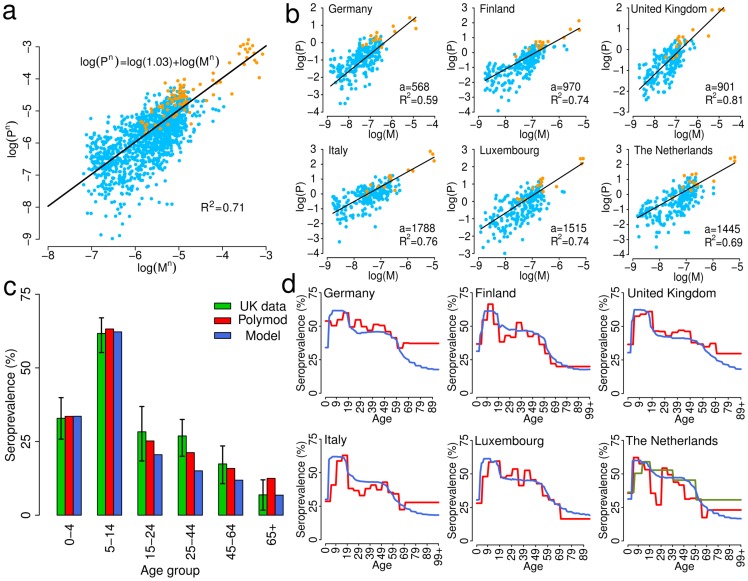
Comparison with Polymod contact matrices. **a** Linear regression model with zero intercept for Polymod matrices [9] 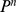 against those from our model, 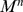 (results shown in logarithmic scale). All countries are considered together and every matrix is normalized so that the sum of its elements is one. Yellow dots refer to the terms on the diagonal, light blue dots correspond to the other entries of the matrices. The value for the regression coefficient is 1.03 and the coefficient of determination 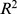 results to be 0.71. **b** As in **a** but for each country singularly, without matrix normalization. In every plot the values for the regression coefficient 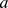 and the coefficient of determination 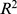 are reported. **c** Green bars represent the average seroprevalence of H1N1 influenza infections in England and Wales during the 2009 pandemic as estimated in a serosurvey [49] (in that study a titre 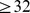 for haemagglutination inhibition has been considered for defining seroconversion in the population) and the black lines represent the 95%CI. Blue bars represent the seroprevalence as obtained by simulating a SIR model with 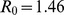 using our contact matrix. Red bars represent the seroprevalence as obtained by simulating a SIR model with 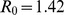 using the Polymod contact matrix. **d** Simulated seroprevalence profiles by age. using Polymod (red) and our matrices (blue), for an epidemic emerging in a completely susceptible population, assuming 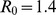. In the plot for the Netherlands the profile obtained using the matrix from [7] is also shown (dark green).

In order to validate the data driven modeling approach at the origin of the synthetic contact matrices we compared our matrices with those obtained by the Polymod project [9]. Polymod currently represents the most accurate and extensive study on mixing patterns in Europe. We considered only the subset of countries common to both our approach and the survey study, namely Germany, Finland, United Kingdom, Italy, Luxembourg and the Netherlands. By jointly regressing the matrices for all countries, each of them normalized so that the sum of all its elements is one, the value of the coefficient of determination 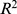 for the linear regression model is 0.72. However, we found that the estimated value of the intercept is very close to 0 (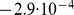, *p*-value = 0.01). Thus we fit a linear model with zero intercept and a single slope coefficient (Figure 4a), concluding that most statistical variation between Polymod matrices and ours can be captured by a single scale factor. Taking every country singularly and applying the linear model with zero intercept to the original matrices (without normalization), we got a coefficient of determination ranging from 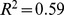 in Germany to 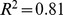 in the United Kingdom (Figure 4b). The estimated scale factor depends on the considered country, because of the large variability in the average number of contacts of an individual in the Polymod matrices. A further comparison highlighting the similarities between Polymod and our contact matrices is shown in Text S1.

### Contact matrices and seroprevalence profiles

Another way to assess and validate our approach consists in the analysis of the prevalence by age profile generated by using Polymod matrices and our synthetic matrices in ILI epidemic models. As an example, we considered the epidemic prevalence generated by an age structured SIR model in a fully susceptible population (as detailed in the Materials and Methods section). The model includes the heterogeneity of contacts by age by introducing a force of infection across age groups modulated by the matrix 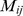. We considered a basic reproduction number 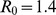 and compared the results obtained by using the contact matrices derived from both the Polymod survey and our model. The results reported in Figure 4d show that Polymod matrices and synthetic matrices yield qualitatively comparable profiles of seroprevalence by age for all countries, with a few noticeable deviations in Germany and the Netherlands (i.e. the countries having the lowest values of 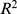 for the linear model, see Figure 4b). As a double check with regards to the Netherlands, we performed the simulations also by considering the contact matrix reported in [7] and obtained by a survey prior to the Polymod one. In this case the results are in good agreement for age groups under 60 years old, while for elderly individuals our matrix gives results closer to those obtained by using the Polymod matrix.

Furthermore, in order to validate numerical simulations against empirical data, we compared predictions of our and Polymod contact matrices to seroprevalence data collected in England and Wales at the end of the second wave of the 2009 H1N1 pandemic influenza [49]. We simulated this epidemic by using both the Polymod and our contact matrix for the United Kingdom. An age-specific susceptibility to infection was assumed; however, this parameter has not been fitted to epidemic data, but its value has been set to 2.0 for children under 16 years of age, following the estimate in [50] later confirmed in [5], [51]. The only degree of freedom in the fit is therefore represented by the scale factor which can be tuned to obtain different values of 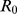. The results are shown in Figure 4c: our model is able to reproduce well the seroprevalence of individuals in the classes 0–4, 5–14 and 65+ years old, while it underestimates intermediate age groups, where the Polymod matrix instead performs slightly better. Overall, model simulations belong to the 95% confidence interval of serological data in five of the six age groups considered in [49].

It is worth remarking that profiles predicted by employing our matrices are smooth because the proportions of contacts are derived from the entire simulated population. Polymod matrices instead are based on the observation of a sample of the population, and this leads to a less regular seroprevalence profile (as can be seen for instance for the Netherlands, where prevalence for individuals aged 19–29 appears to be much lower than for the adjoining age groups). More in general, the prevalence predicted from the synthetic mixing patterns is higher among school-age children, intermediate for working ages and progressively declining in the elderly; prevalence among little children is at an intermediate level. This pattern is mainly driven by country-specific employment and schooling rates, along with the scholastic organization. Simulated seroprevalences, using our contact matrices in the same epidemic setting, for the countries not covered by Polymod are provided in Text S1. The shapes are all similar; however, some differences are visible: for instance, following the decline of prevalence after school age, a second steep decay occurs in all countries at a variable age, generally higher (around 60) in Northern Europe and lower (around 50) elsewhere and more markedly in Southern countries. This is probably an effect of the differences in the retirement age across Europe, as we previously pointed out.

### Comparison with average European matrix

An intermediate choice between homogeneous mixing and country-specific contact patterns would be to consider mixing patterns as derived by appropriately averaging over the 26 country-specific contact matrices; therefore in this section we compare results obtained by assuming homogeneous mixing, the average European matrix and country-specific matrices. We considered an SIR model where all the basic parameters and scaling factors are set on the baseline yielding a basic reproduction number 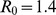 for the European average. For each country we used the synthetic contact matrices aggregated by one-year age brackets up to 84 years of age (so that all matrices have the same dimension). All other parameters being equal, the different contact matrices in each country define different values of 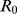 and different epidemic behaviors in each country. In particular, the reproduction number 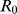 can be calculated (see Materials and Methods section) for each contact matrix.

**Figure 5:**
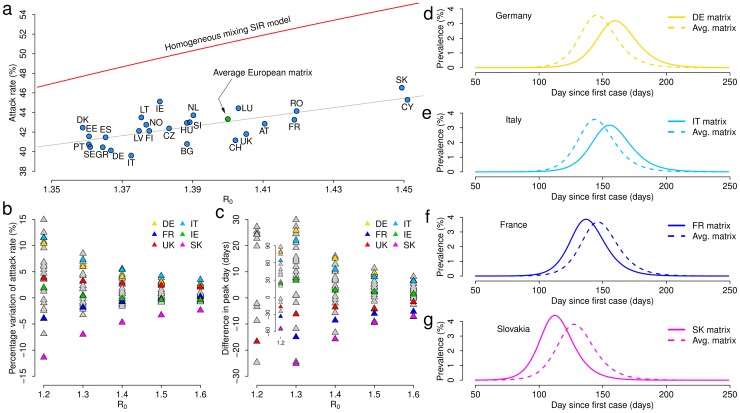
Country-specific matrices and European average. **a** Final infection attack rate as a function of the basic reproduction number 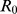 in the different countries (blue dots) by adopting country-specific matrices and by assuming the same probability of transmission 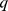 in all countries – specifically, the value resulting in 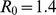 by adopting the average European matrix (green dot). The attack rate corresponding to the average European matrix is computed by assuming the average European age structure in the model. Red line represents the attack rate of the homogeneous mixing SIR model for values of 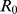 in the range of variability of the basic reproduction number of country-specific matrices. Grey line represents the best fit of the linear model to data points related to the use of country-specific matrices. **b** Percentage variation of infection attack rate for increasing values of 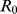 of models based on country-specific matrices with respect to models based on the average European matrix (with country-specific age structure). **c** As **b** but for the variation of the peak day. **d–g** Daily prevalence over time of models with 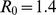 based on either the country-specific matrix (solid lines) or the average European matrix (dashed lines, with country-specific age structure) in Germany, Italy, France and Slovakia respectively. In this figure we assume the generation time to be 3.1 days.

The improvement obtained by using either the European average matrix or country-specific matrices compared to the homogeneous model is evident e.g. in terms of final attack rate which, as already noticed in previous computational studies [26], is always overestimated by the homogeneous assumption (Figure 5a). Although we found a strong positive correlation between 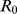 and attack rate following the country-specific approach (Pearson correlation test 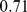, *p*-value 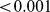), the result is clearly far from the homogeneous mixing model that does not consider any contact structure.

By applying to every country the average European contact matrix, large differences in terms of attack rate and peak day can be observed compared to the results obtained with the country-specific mixing patterns, especially for values of the basic reproduction number consistent with influenza epidemics (Figures 5b–c). These variations are driven only by the structure of contacts used, as the population considered is the same. In particular, depending on 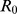, peak days may differ by several weeks (Figure 5c). This is clear also from Figures 5d–g, where epidemic profiles corresponding to 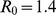 for four countries are shown. Moreover, the use of the average contact matrix yields a general alignment of epidemics in the different countries, while differences in timing are clearly visible when country-specific matrices are used.

### Socio-demographic structure and disease epidemiology

The synthetic contact matrices allow us to analyze the effect of the different social and demographic structure of countries on the evolution of infectious diseases characterized by the same natural history. For the sake of simplicity we considered an SIR model with basic parameters and scaling factors corresponding to 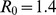 in the UK. In each country we used the synthetic contact matrices aggregated by 5-year age brackets up to the class 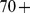.

**Figure 6:**
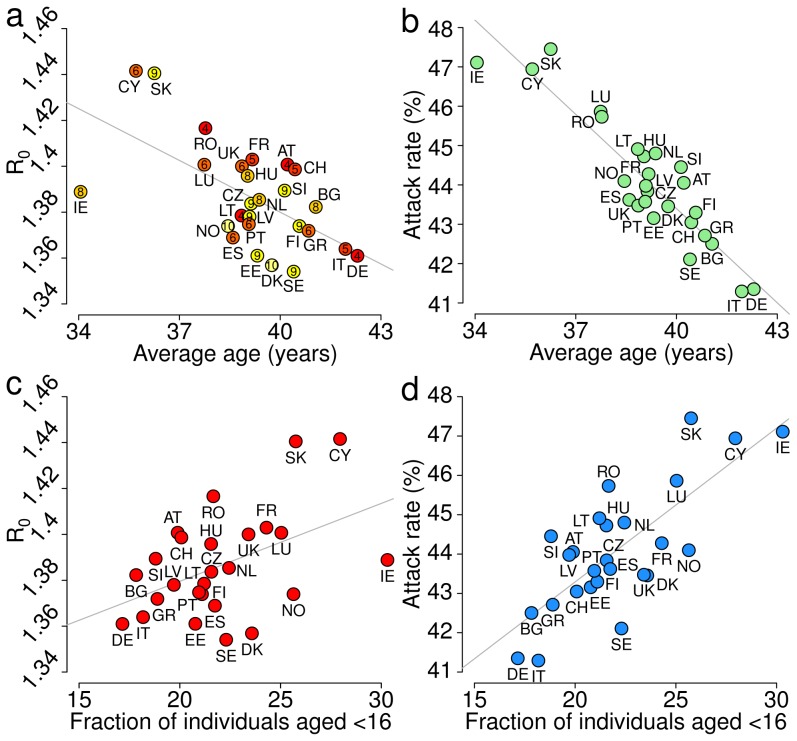
Socio-demography and disease epidemiology. **a** Basic reproduction number 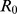 as a function of the average age of the population in the different countries. Numbers inside the circles represent the duration (in years) of the primary school cycle; colors from red to yellow are proportional to those numbers. **b** Final attack rate as a function of the average age of the population in the different countries. **c** Basic reproduction number as a function of the fraction of individuals younger than 16 years of age in the different countries. **d** Final attack rate as a function of the fraction of individuals younger than 16 years of age in the different countries.

The average age of the population is the single factor best explaining the basic reproduction number (correlation 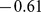, *p*-value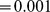; and the linear regression model with the average age as the only independent variable gives a coefficient of determination 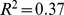, *p*-value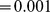). We found that a linear model having both average age and matrix assortativeness (measured by the *Q*-index [11], see Text S1) as independent variables represents the best option to explain the variability of 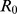 between countries: 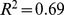, *p*-value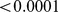. However, matrix assortativeness cannot be derived directly from routinely collected data, but is characteristic of the specific contact matrices, therefore it is unknown *a priori*. Nonetheless, matrix assortativeness is strongly related to the duration of the primary school cycle (correlation 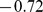, *p*-value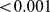). Therefore, we decided to add the duration of the primary school cycle as a proxy for matrix assortativeness in the linear model for explaining 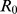 having average age as independent variable; this model gives 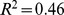 and the analysis of variance reveals a statistically significant improvement (*p*-value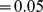) with respect to the model considering the average age as the only independent variable (see Figure 6a). As regards the final attack rate, we found that the best single socio-demographic factor explaining the variability between countries is the average age of the population (correlation 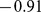, *p*-value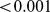; see Figure 6b). Finally, less strong correlations between socio-demographic features of the populations and epidemiologically relevant quantities are shown in Text S1. We also found a relationship between the proportion of individuals less than 16 years old in the population and the basic reproduction number (correlation 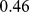, *p*-value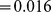; Figure 6c), in line with a recent study on the 2009 H1N1 influenza pandemic [55]; the correlation of this fraction of the population with the attack rate is even stronger (correlation 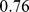, *p*-value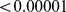; Figure 6d). These results highlight that the epidemic spread is affected by the presence of younger individuals in the population, who are generally exposed to a larger force of infection than the elderly.

### Conclusion

In this work we propose a method, based on the analysis of the contact network in a highly detailed virtual society, and compute the related matrices of adequate contacts for 26 European countries.

Our analysis highlights well defined correlations between epidemiological parameters and socio-demographic features of the populations. Specifically, we found that the basic reproduction number is well explained by a linear model having average age of the population and duration of primary school cycle as independent variables, whose values are easily derivable from routinely collected social and demographic data. In addition, the average age appears as the main determinant in explaining differences in final attack rates between countries. In this perspective, the use of synthetic contact matrices helps in improving the accuracy of mathematical models predictions, which are increasingly used for supporting public health decisions.

It is worth remarking that the presented approach is based on routinely collected data, and it can be easily extended to every country for which socio-demographic data are available. Notably, by providing information by one-year age brackets, our contact matrices are particularly suitable when dealing with childhood diseases which require detailed information on contact patterns in the youngest age classes. Finally, our method may be used also retrospectively, in order to reconstruct contact patterns in the past by using data from previous census rounds; this would be useful to review classic results based on indirect estimates of contacts, such as WAIFW matrices [47].

## Supporting Information

Table S1 **Total matrices of adequate contacts.** Frequencies of total contacts by age for 26 European countries. Please note that disease transmission models usually require the average frequencies of contacts by age; these can be obtained by dividing the symmetric matrices given in this table by the age structure of the population.
(XLS)

Table S2 **Setting-specific contact matrices.** Matrices of contacts in households, schools, workplaces and in the general community for 26 European countries. Please note that disease transmission models usually require the average frequencies of contacts by age; these can be obtained by dividing the symmetric matrices given in this table by the age structure of the population.
(XLS)

Text S1 **Supporting text.** Supporting text containing methodological details and additional results.
(PDF)
