# Supplementary material for: Inferring the Structure of Social Contacts from Demographic Data in the Analysis of Infectious Diseases Spread
Source: PLoS Comput Biol. 2012 Sep 13;8(9):e1002673. doi: 10.1371/journal.pcbi.1002673 (PMC3441445; doi:10.1371/journal.pcbi.1002673)
Supplement: Text S1 — Supporting text. Supporting text containing methodological details and additional results. (PDF) [file pcbi.1002673.s003.pdf]

Inferring the Structure of Social Contacts from Demographic Data in the Analysis of Infectious Diseases Spread

Laura Fumanelli[1], Marco Ajelli[1], Piero Manfredi[2], Alessandro Vespignani[3,4,5] & Stefano Merler[1]

[1] Bruno Kessler Foundation, Trento, Italy,
[2] Department of Statistics and Mathematics Applied to Economics, University of Pisa, Italy
[3] Department of Health Sciences and College of Computer and Information Sciences, Northeastern University, Boston, MA
[4] Institute for Quantitative Social Sciences at Harvard University, Cambridge MA
[5] Institute for Scientific Interchange Foundation, Turin, Italy

Text S1

Contents

1 Materials and methods **2**
1.1 Households . . . 2
1.2 Schools and workplaces . . . 8
1.3 Contact matrices . . . 11

2 Clustering and networks **16**

3 Comparison with Polymod **17**

4 Simulation of a pandemic event **22**

5 Socio-demographic structure and disease epidemiology **23**

1 Materials and methods

We simulate a virtual society of synthetic individuals in order to derive contact matrices for all member states of the European Union except Belgium, Poland and Malta, and together with Switzerland and Norway, for a total of 26 countries (see Table S1). The total population of the study area is more than 4 hundred millions, the most populous country being Germany ($\approx$ 81 millions inhabitants), while the less populous one is Luxembourg ($\approx$ 310 thousands).

1.1 Households

We use a heuristic model matching marginal distributions of household age by size, age of household members by size (and thus the age structure of the total population), and maintaining

**Label**	**Country***	**Population**
AT	Austria	8,294,168
BG	Bulgaria	8,133,261
CH	Switzerland	7,061,889
CZ	Czech Republic	10,363,160
DE	Germany	81,868,705
DK	Denmark†	5,286,833
ES	Spain	38,243,265
EE	Estonia	1,389,529
FI	Finland	5,128,201
FR	France	59,420,100
UK	United Kingdom	59,261,769
GR	Greece	10,779,882
HU	Hungary	9,853,889
IE	Ireland	3,821,683
IT	Italy	57,325,816
LT	Lithuania	3,721,772
LU	Luxembourg	311,515
LV	Latvia	2,402,695
NL	The Netherlands	15,118,219
NO	Norway	4,393,341
PT	Portugal	9,554,549
RO	Romania	22,099,494
SK	Slovakia	5,392,743
SI	Slovenia	2,164,774
SE	Sweden	8,818,111
CY	Cyprus	770,956
–	**Total**	**440,980,319**

* Overseas territories excluded.

† Greenland and Faroe Islands excluded.

Table S1: Countries of the study area. Number of individuals in 2008 as obtained from Eurostat database [1].

realistic generational age gaps within household members. Data on number of people by age and household size, on frequencies of household size and household type are provided by the Statistical Office of the European Commission (Eurostat) [1] and refer to year 2001. Since data on household age by size are available for 5-year age groups, at this stage we assign to individuals an age class from 0 (0-4 years old) to 20 (100+ years old).

The procedure used to build a household is the following:

1. determine the size by sampling from the distribution of household size;

2. assign an age a_h to the household head, by sampling from the distribution of age classes for the specific household size, under the constraint that $a_h \geq 3$ (i.e., ≥ 15 years old);

3. for households having two, three or four members, determine if there is a single adult or a couple, according to the corresponding probability for households of the assigned size. For the sake of simplicity, we do not consider households with more than two adults, e.g. a couple with children and an aggregate member. All households with more than four members are assumed to be composed by a couple with children, since type "couple with children" represents more than 95% of the total number of households;

4. assign an age to the other members by sampling from the distribution of age classes for the specific household size, taking into account the following constraints:

   (a) the age of the (possible) spouse, a_s, satisfies $\max\{a_h - 3, 3\} \leq a_s \leq \min\{20, a_h + 3\}$;

   (b) the age of (possible) children, a_c, in a household with a single adult, satisfies $\max\{0, a_h - 8\} \leq a_c \leq a_h - 4$;

   (c) the age of (possible) children, a_c, in a household with two adults, satisfies $\max\{0, a_m\} \leq a_c \leq \min\{a_h, a_s\} - 4$, where $a_m = \max\{a_h, a_s\} - 8$.

A large variability in frequencies of households size is observed among the considered countries (see Figure S1): Ireland, Cyprus and Romania have a remarkable (compared to the other countries) fraction of households with seven or more components, while households with one individual are more common across Central and Northern Europe. The observed distribution of household size is accurately reproduced by the model, as well as the average values (Figure S1 and S2): it can be noticed that, in line with the previous remark, countries in Central and Northern Europe are characterized by a lower average size.

The distribution of individuals by age group for a given household size differs from country to country: Figure S3 compares real and simulated structures for four countries, namely those with the highest and lowest average age (Germany and Italy on one hand, Ireland on the other) and an intermediate situation (United Kingdom). The proportions of household sizes (represented by the grey dots on the left) are not the same for all countries, for instance the proportion of households with seven or more members in Ireland is much higher than in the other countries. In all countries, in households with three members or more the age distribution shows two peaks, corresponding to children and parents. Notably, we observe a variability in terms of age structures: for instance, in Italy most single-member households consist of an elderly individual; this is slightly visible also for Germany, while in Ireland and United Kingdom this is much less evident; moreover, in the latter countries a much larger proportion of members of households of size two is under 30 years of age. The composition of households with seven or more members,

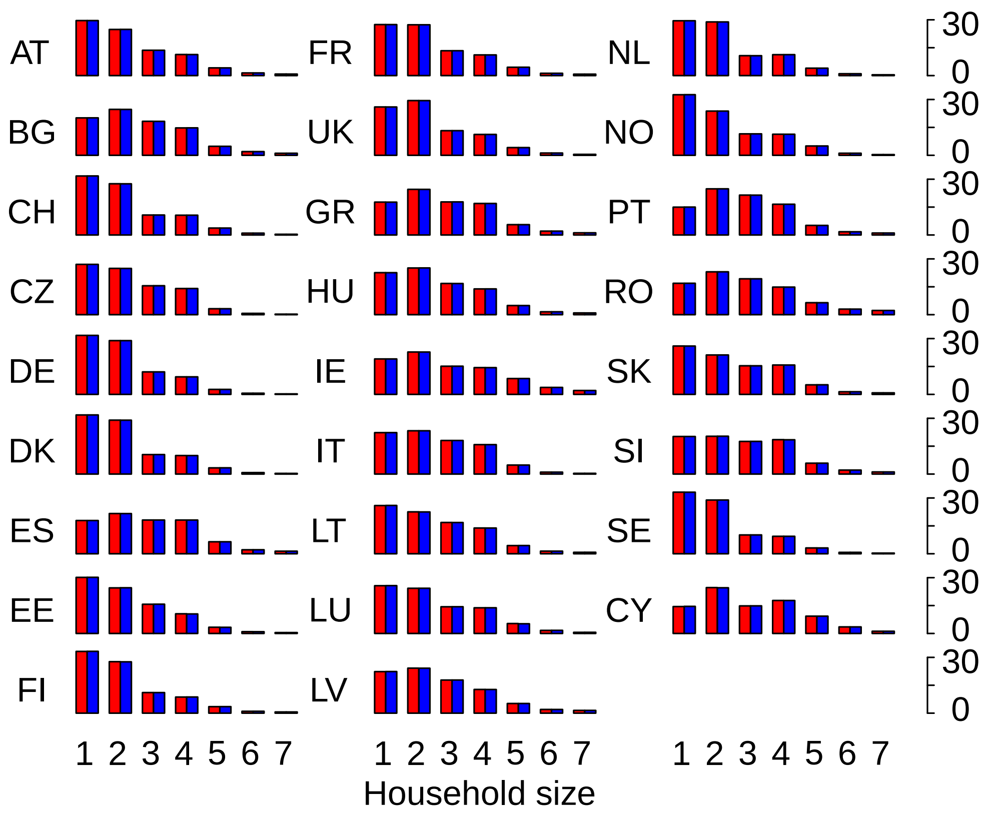

Figure S1: Percentage of household size, real (red) and simulated (blue), for all countries of the study area.

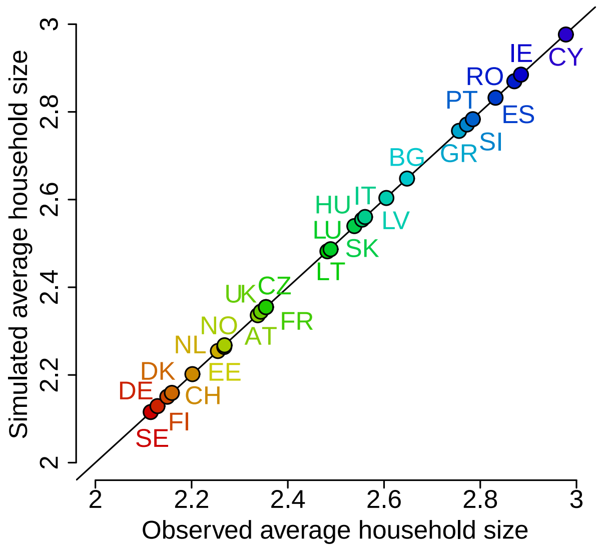

Figure S2: Comparison between simulated and observed average household size.

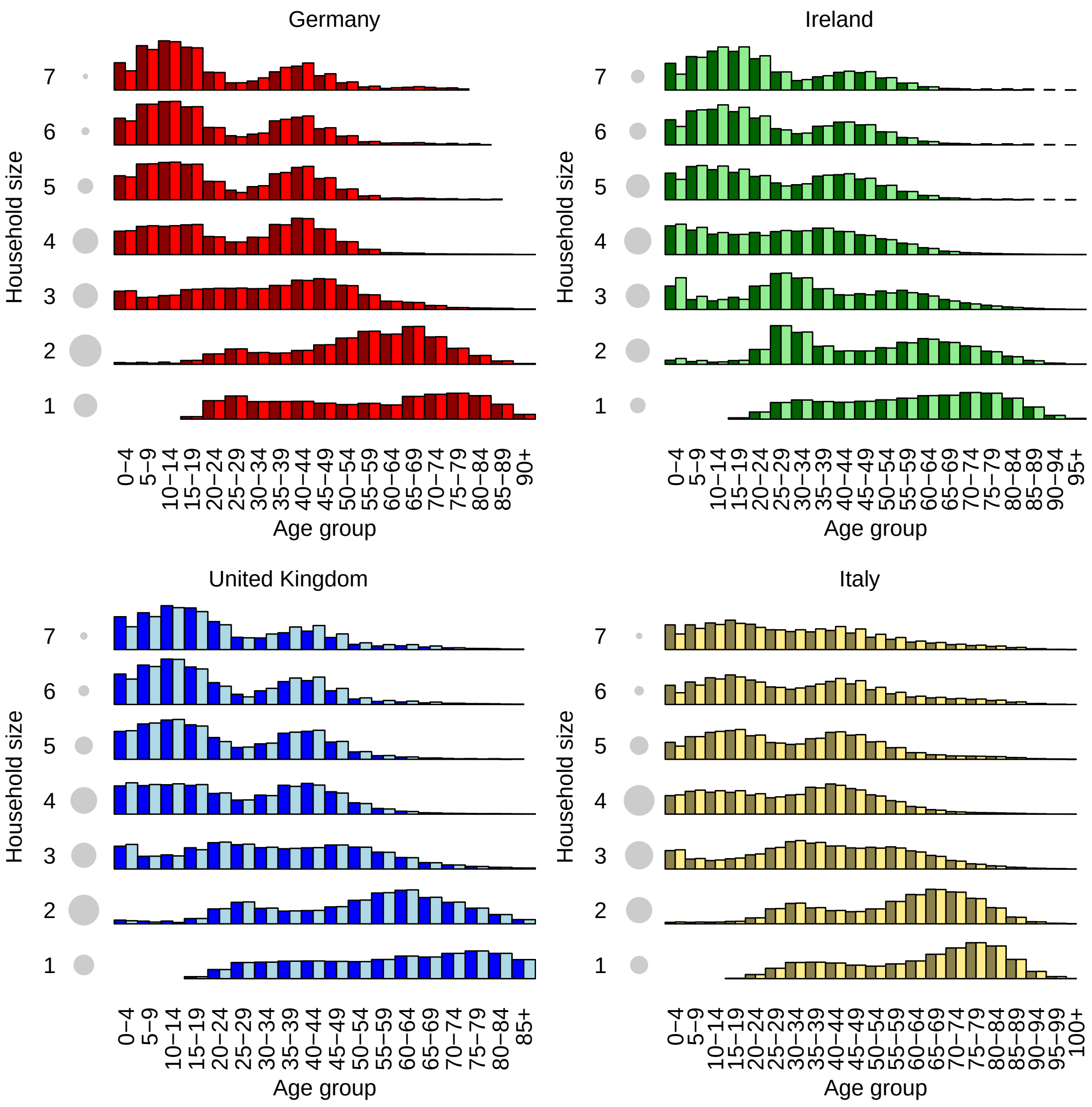

Figure S3: Age structure by household size in Germany, Ireland, United Kingdom and Italy. Dark colors represent real data, light colors refer to simulated data. The area of the grey circles on the left of every distribution is proportional to the fraction of households of the specific size for the considered country.

with many children and middle-aged adults, is visible in all countries except Italy, where a relatively high proportion of young adults lives in big households.

Simulated age structures by household size comply well with observed data (see FigureS3); this supports the validity of our choice on household types: neglecting non-private households or compositions such as families with aggregated members does not lead to significant differences between real and simulated households structure.

Overall, simulated households comply very well with real data, in terms of average size (Figures S1 and S2), distribution of age groups given the size (Figure S3) and type of composition (Figure S4).

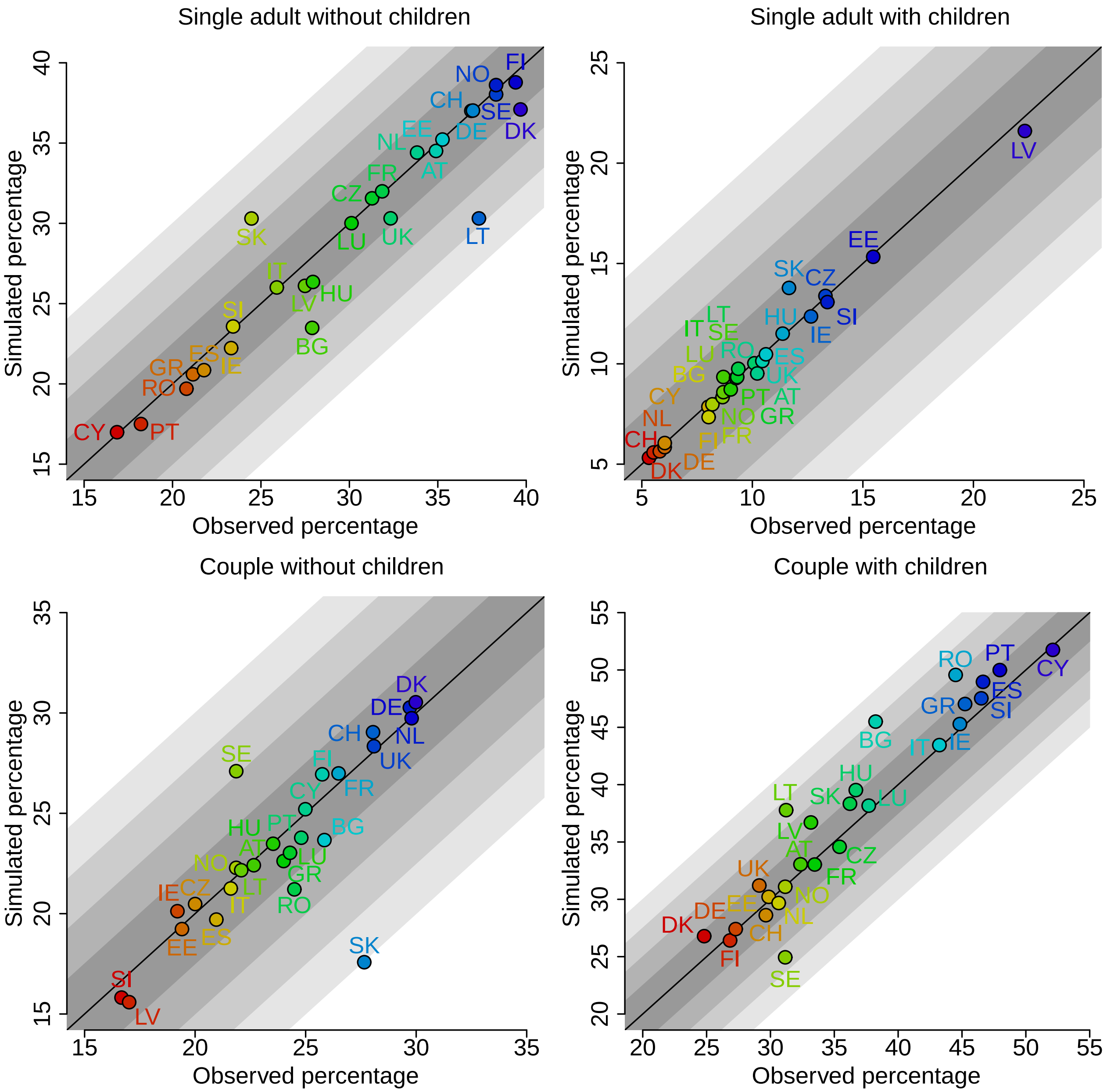

Figure S4: Comparison between simulated and observed household types percentages for all countries of the study area.

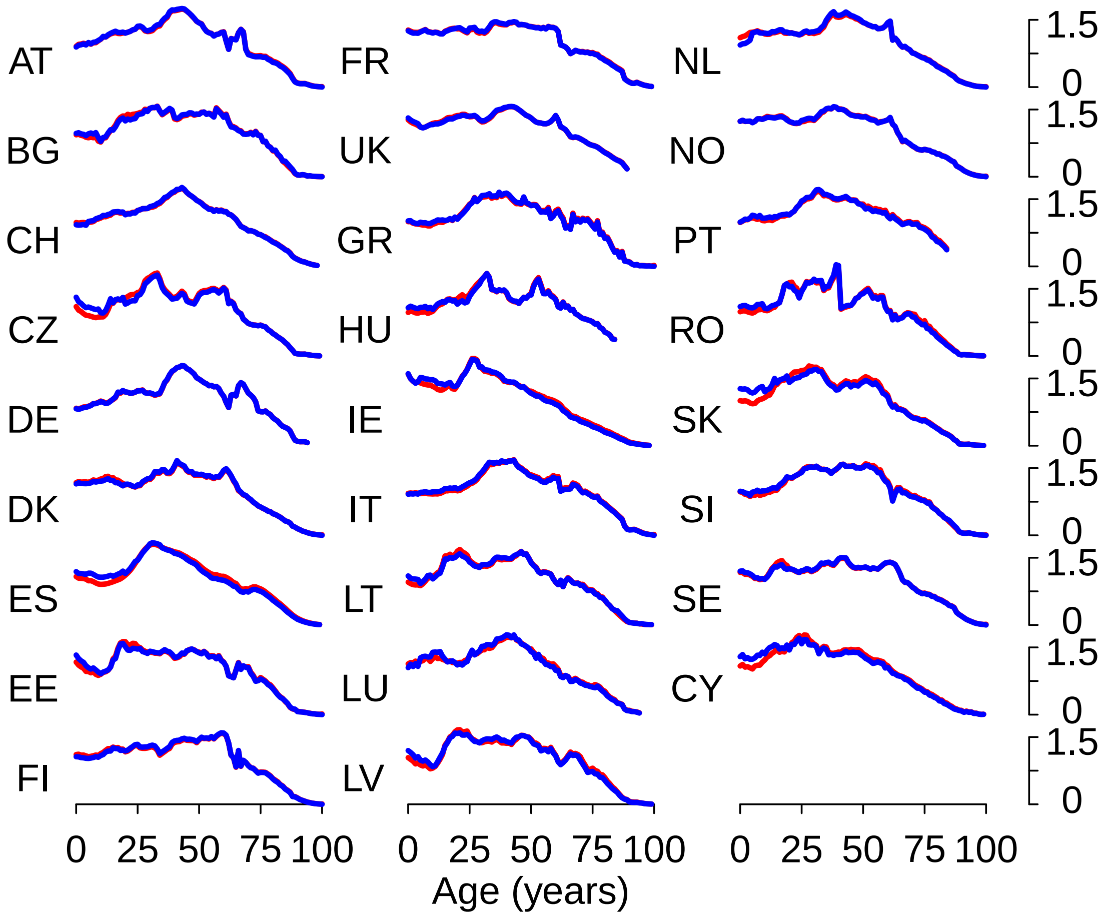

Figure S5: Percentage distribution of individuals of a given age as observed in the data (red) and simulated by the model (blue) for all 26 countries of the study area.

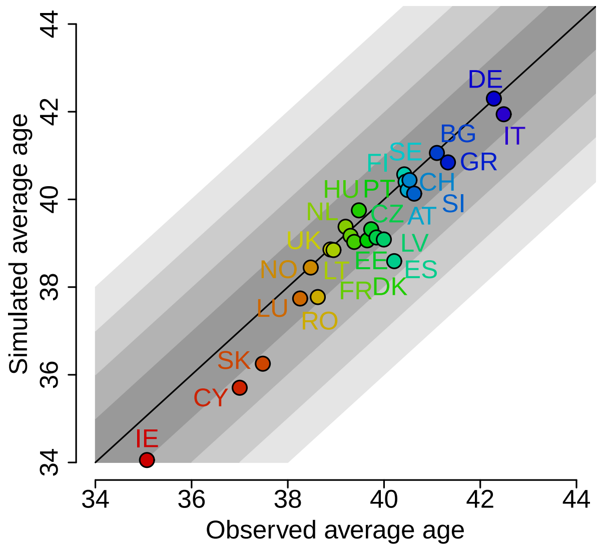

Figure S6: Comparison between simulated and observed average age for all 26 countries.

1.2 Schools and workplaces

An occupation is then assigned to individuals; more precisely, every member of the population either goes to school (as a student or teacher/school employee) or workplace, or remains at home (for instance as a retired or family worker).

To evaluate the frequencies of the different occupations by age, we use country-specific 2001 census data from Eurostat [1] on the number of active and inactive (i.e., not belonging to the labour force) individuals; the latter status comprises people in education, which we consider separately. For this category we use 2008 Eurostat data on the number of students and on the total population by age. More recent data on rates of activity/inactivity are not available to us. Unfortunately, these data are not provided at all for Belgium, Malta and Poland, and this is the reason why we cannot include these countries in our study: this information is crucial in determining contact patterns, hence using average data as obtained from other countries to fill the gap would be an unsuitable choice.

Due to the type of available data, first of all we switch from the 5-year age groups used when building households to a year-by-year age structure: an age between 0 and 100+ is attributed to individuals, according to the age group they belong to after the first assignment and to the

**Label**	**Country**	**Primary**	**Lower secondary**	**Upper secondary**	**Higher education**	**Unique cycle**
AT	Austria	6	10	14	18	no
BG	Bulgaria	7	-	15	19	yes
CH	Switzerland	7	12	15	19	no
CZ	Czech Republic	6	-	15	19	yes
DE	Germany	6	10	16	19	no
DK	Denmark	6	-	16	19	yes
ES	Spain	6	12	16	18	no
EE	Estonia	7	-	16	19	yes
FI	Finland	7	-	16	19	yes
FR	France	6	11	15	18	no
UK	United Kingdom	5	11	14	18	no
GR	Greece	6	12	15	18	no
HU	Hungary	6	-	14	18	yes
IE	Ireland	4	12	15	18	no
IT	Italy	6	11	14	19	no
LT	Lithuania	7	11	17	19	no
LU	Luxembourg	6	12	15	19	no
LV	Latvia	7	-	16	19	yes
NL	The Netherlands	4	12	15	18	no
NO	Norway	6	-	16	19	yes
PT	Portugal	6	12	15	18	no
RO	Romania	6	10	16	19	no
SK	Slovakia	6	-	15	19	yes
SI	Slovenia	6	-	15	19	yes
SE	Sweden	7	-	16	19	yes
CY	Cyprus	6	12	15	18	no

Table S2: Age (years) at which children enter the different school cycles [2, 3]. The organization of primary and lower secondary schools into a single structure is highlighted in the last column.

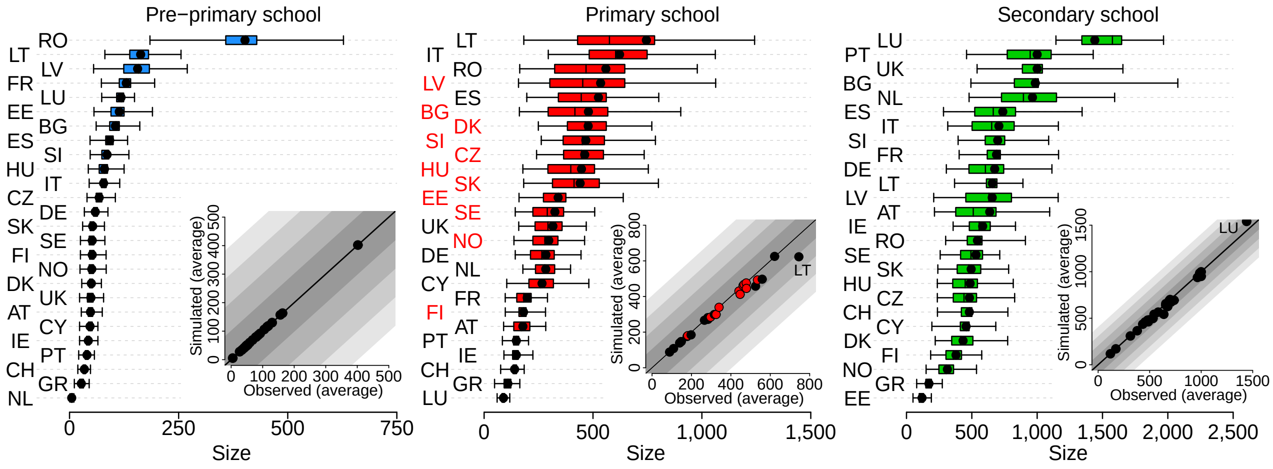

Figure S7: Boxplots (representing 2.5, 25, 50, 75 and 97.5 percentiles) of the size of simulated pre-primary (blue), primary (red) and secondary (green) schools in the different countries of the study area. Black dots are the observed average school sizes. Red labels in the panel for primary schools identify countries where primary and lower secondary schools are organized as a single structure. In the insets a comparison between simulated and observed average school size for pre-primary, primary and secondary schools is shown. Red dots in the panel for primary schools represent countries where primary and lower secondary schools are organized as a single structure.

observed distribution of corresponding ages. For example, if we consider an individual living in country r and belonging to age group 4 (i.e., 20-24 years old), we assign her/him an age by sampling from the age structure of country r in the range between 20 and 24 years. The simulated age structures are in good agreement with the observed ones for all 26 countries (see Figure S5 and S6).

Moreover, refining age allows us to assign the correct school level to students. The educational system is generally divided into five levels: pre-primary (day-care centers, kindergartens), primary, lower secondary, upper secondary, higher education (post-secondary training, university, doctoral programs); these stages do not always correspond to those defined by the UNESCO International Standard Classification of Education (ISCED 97) [2, 4]. In several countries (in Northern and Eastern Europe) there is no distinction between primary and lower secondary education, which are organized as a single structure. Transition between school stages takes place at different ages within Europe [2, 3], as summarized in Table S2.

From the above described datasets, for each country we derive rates of school attendance by age to obtain the number of individuals to be assigned to schools; we assume that all children in compulsory age (up to 16 years) attend school, while younger children may also stay home. People from 17 to 34 years old may attend educational structures, go to work, or be inactive; people aged 35 or more can only be workers or inactive.

As regards schools size, for primary and secondary schools we refer to official reports of the Education, Audiovisual and Culture Executive Agency (EACEA P9 Eurydice) of the European Commission [2, 5, 6], or to national statistics offices [7, 8]. Information on lower and upper secondary schools sizes is generally not separate, so we use the same data for both levels. Data on size of pre-primary schools are derived from [6, 8, 9, 10, 11]. As regards universities, we have no information on the average size of buildings (the context relevant for person-to-person

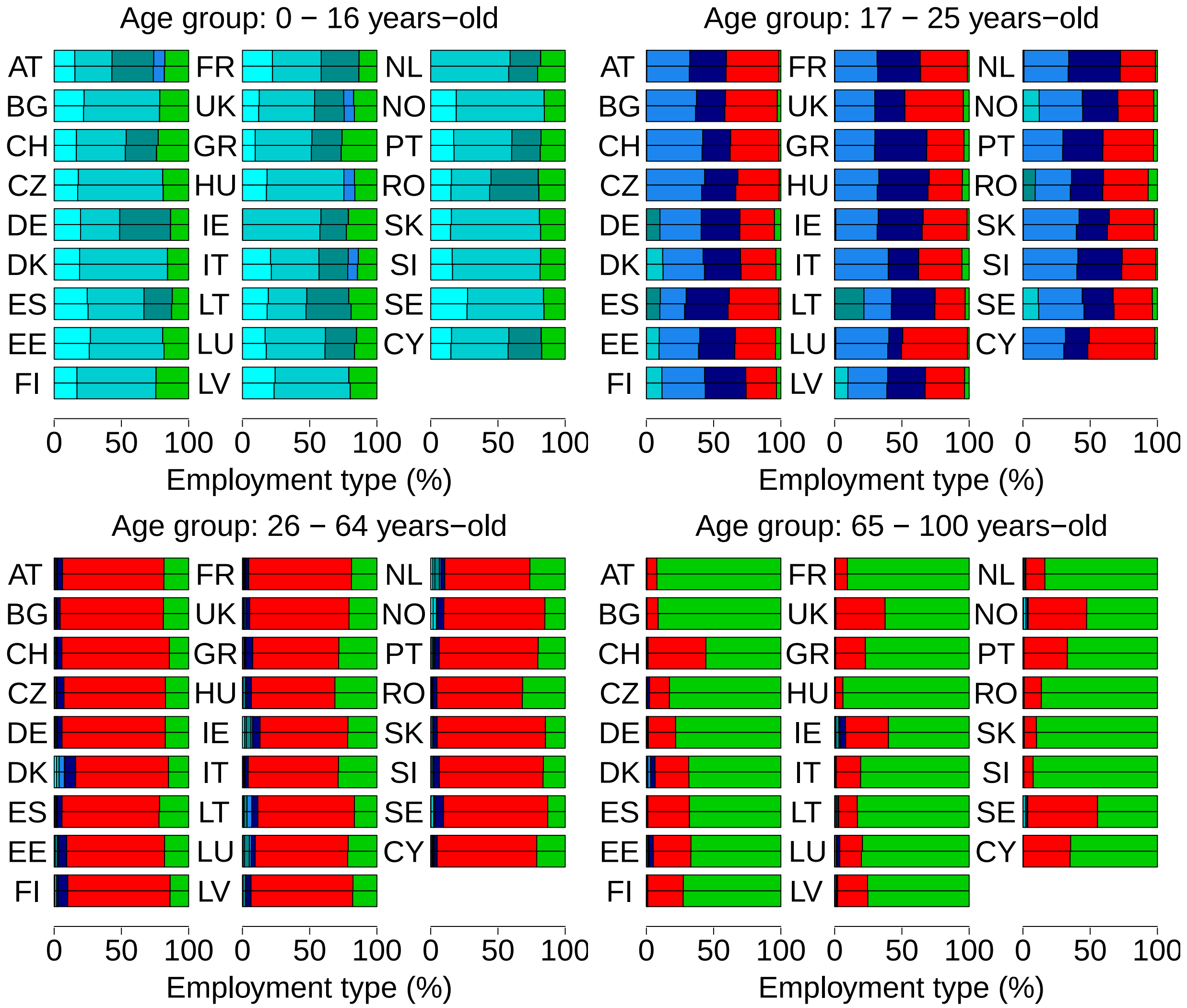

Figure S8: Percentage of individuals by type of employment for age groups 0-16 years old (top left), 17-25 years old (top right), 26-64 years old (bottom left), 65+ years old (bottom right). For each country the upper and the lower bars represent the simulated and observed data, respectively. Colors from light blue to dark blue represent individuals attending the different school levels (as students or teachers); red represents workers (teachers excluded); green represents inactive individuals.

contacts), which are the structures of interest when considering epidemic transmission; therefore we allow the size to vary in a wide range (from 50 to 750).

The overall educational structure is simulated in the model: children and young adults are assigned to schools according to their age and to schools size. With this procedure, sizes of simulated schools agree with observed data for pre-primary, primary and secondary levels (Figure S7); differences among structures within the same country and among countries are clearly reproduced.

Data on workplaces size are not available for all the countries under consideration. However, in [12] it has been shown that no significant differences between the distributions of workplaces size in Italy and in the United Kingdom exist. Therefore, as in [12], these data are combined to determine an average distribution of workplace size, which is used for all simulated countries.

Finally, individuals are assigned to an employment according to their age and to the distribution of workplaces size.

In our model we also consider individuals working as teachers. Based on data on the number of teachers distributed by age and educational level of schools in which they are employed (provided by Eurostat [1]), a fraction of adult workers are assigned to simulated schools.

Individuals in each age class who are neither students/teachers nor workers are considered as inactive.

Distributions of activities by age group as obtained by simulating the model are in good agreement with observed data. Figure S8 shows, for all countries, the real and simulated fractions of individuals attending school (where adults assigned to a school from pre-primary to upper secondary level have to be considered as teachers), working or inactive. Differences between countries with respect to the structure of the educational system and the proportions of students, workers, teachers and inactive are clearly observable.

1.3 Contact matrices

Contact matrices by one-year age brackets for 25 European countries, built as detailed in the Materials and Methods section in the main text, are shown in Figures S9 (households), S10 (schools), S11 (workplaces) and S12 (total matrices of "adequate" contacts). Representations of the matrices for the United Kingdom are shown in Figure 2 of the main text.

A notable characteristic, common to all countries, is the strong assortativeness. In Figure S13 frequencies of same-age contacts for 5-year age classes are reported, for all countries except the United Kingdom (shown in Figure 2f of the main text): a marked tendency of young individuals (corresponding to school ages) to mix mostly with people of the same age can be seen, while this is much less evident in adults and the elderly. Moreover, in countries where primary and secondary schools are organized into a single structure the assortativeness is less marked.

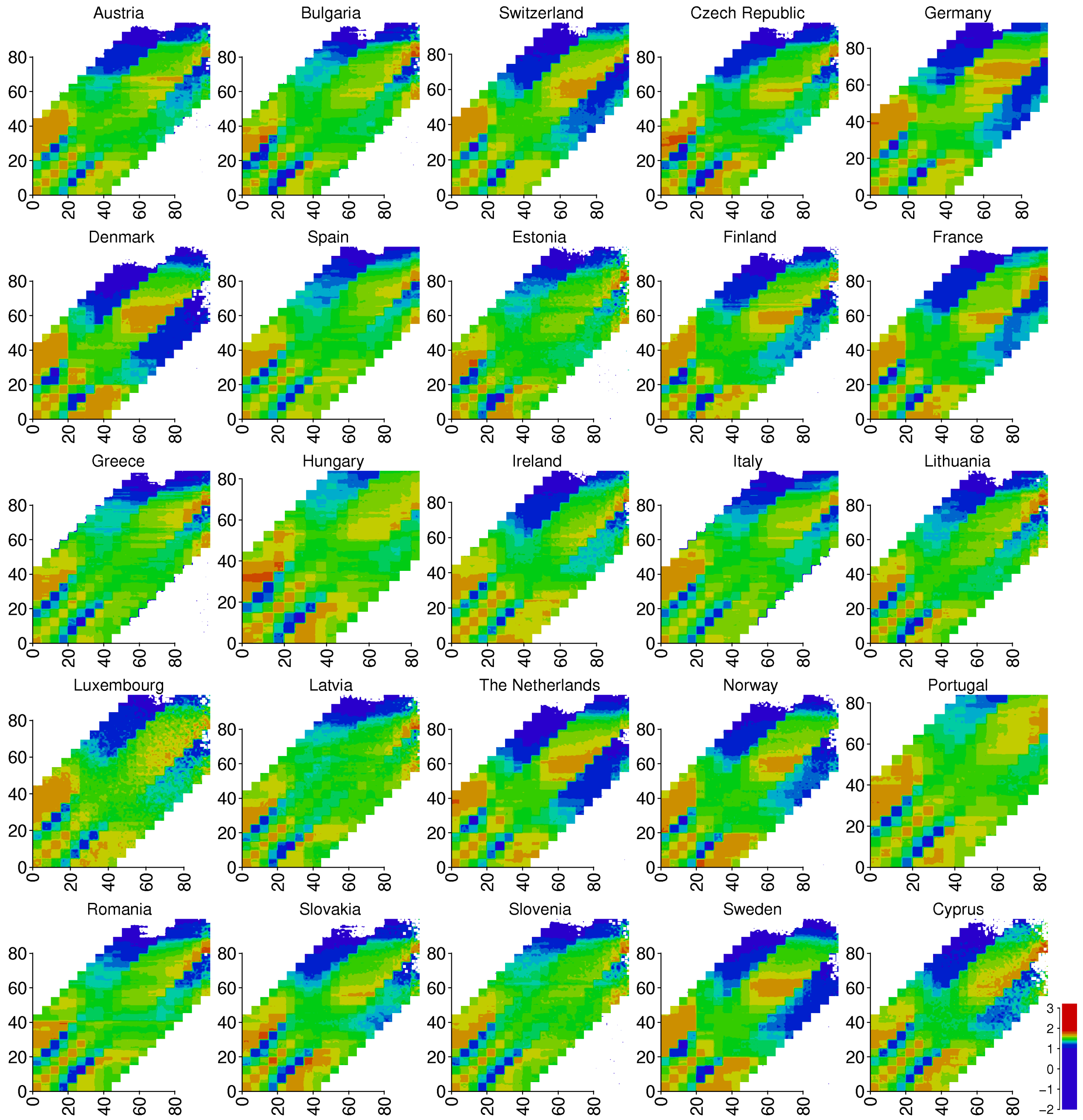

Figure S9: Contact matrices in logarithmic scale by one-year age brackets for contacts within households for all countries of the study area except the United Kingdom (shown in the main text). Frequency of contacts (in arbitrary units) increases from blue to red.

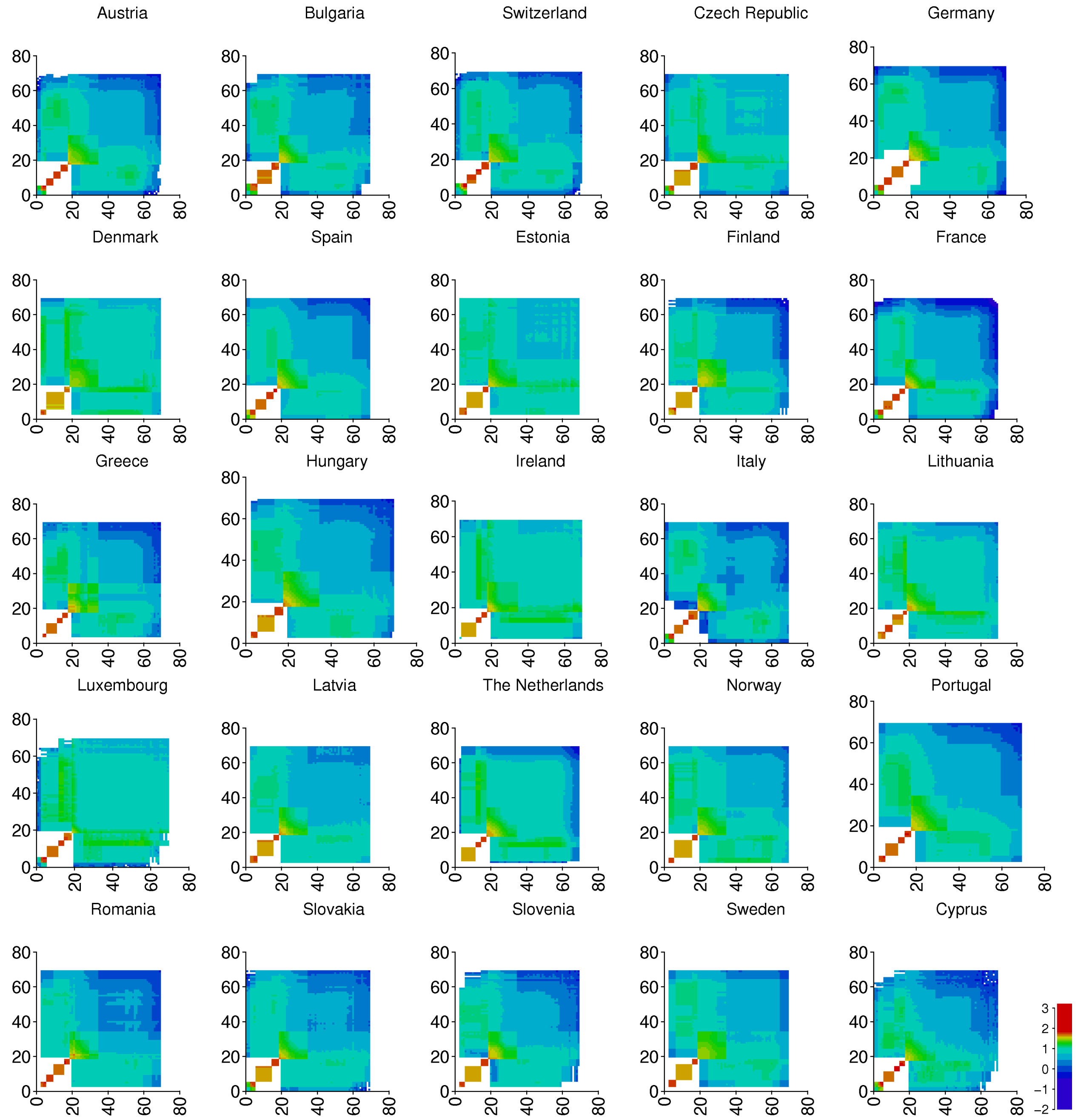

Figure S10: Contact matrices in logarithmic scale by one-year age brackets for contacts within schools for all countries of the study area except the United Kingdom (shown in the main text). Frequency of contacts (in arbitrary units) increases from blue to red.

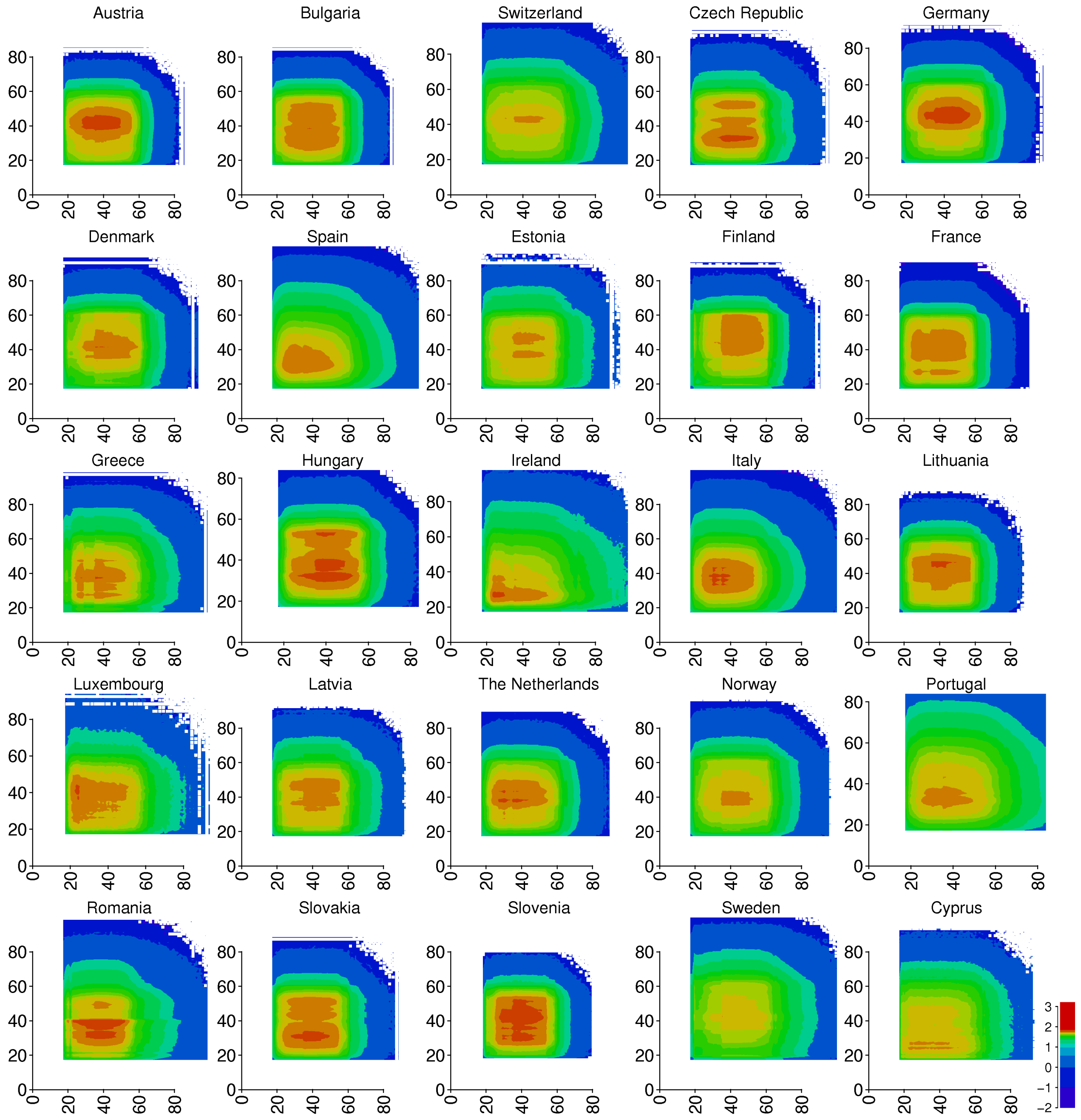

Figure S11: Contact matrices in logarithmic scale by one-year age brackets for contacts within workplaces for all countries of the study area except the United Kingdom (shown in the main text). Frequency of contacts (in arbitrary units) increases from blue to red.

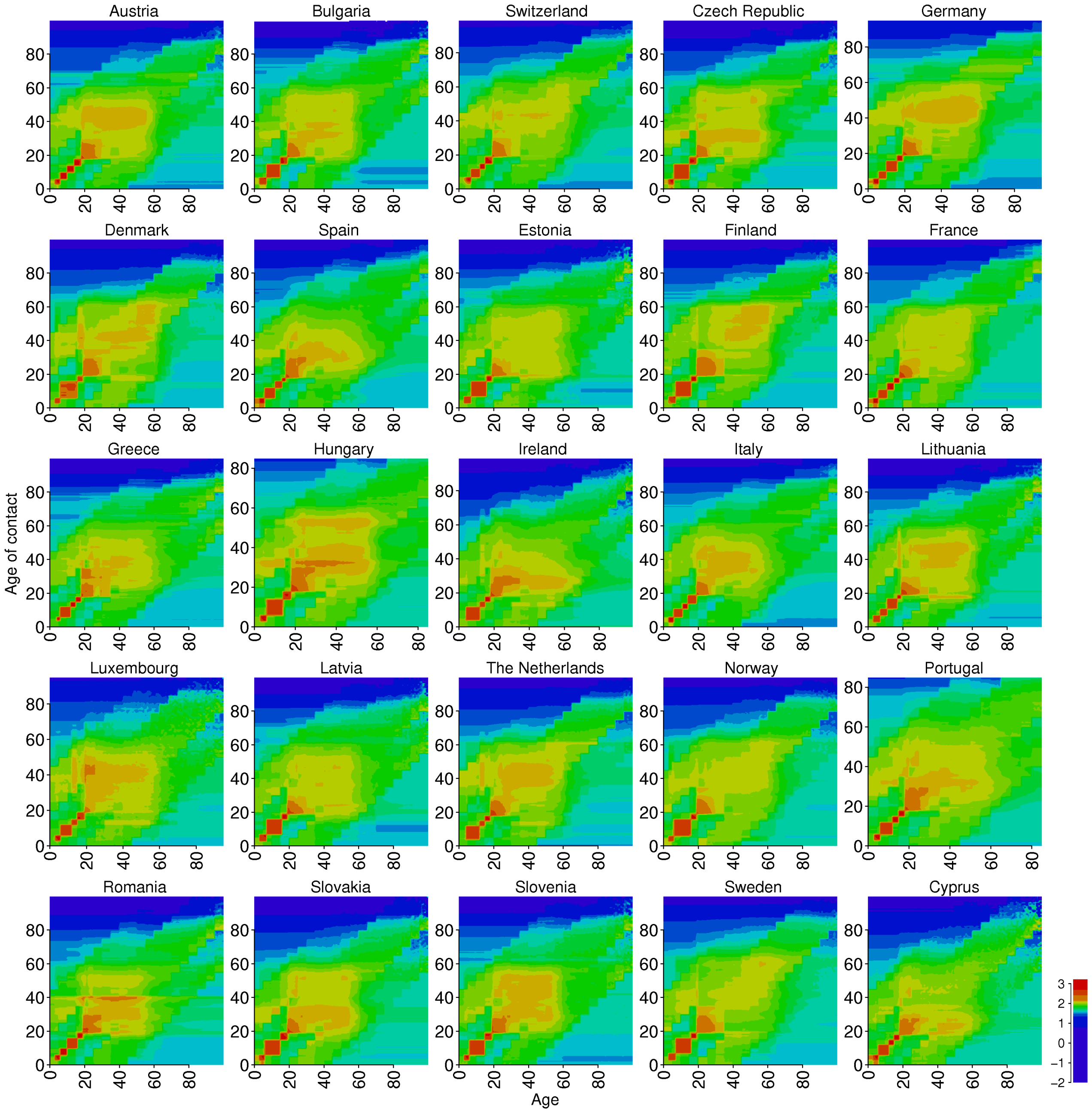

Figure S12: Contact matrices in logarithmic scale by one-year age brackets for total contacts for all countries of the study area except the United Kingdom (shown in the main text). Frequency of contacts (in arbitrary units) increases from blue to red.

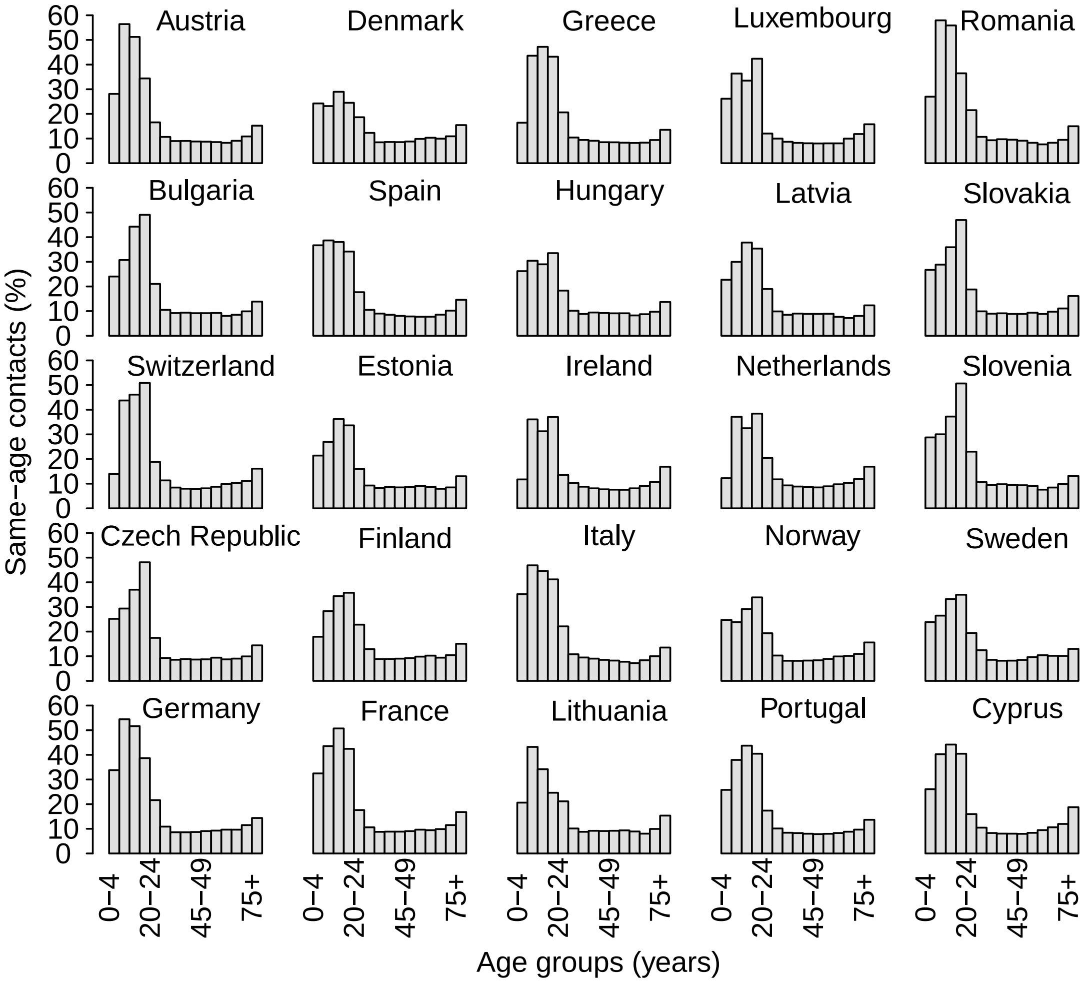

Figure S13: Percentage of same-age contacts for all countries of the study area except the United Kingdom (shown in the main text). Ages are grouped in 5-year classes, except for individuals aged more than 75 who are grouped into a unique class.

2 Clustering and networks

A clustering method was applied to our contact matrices in order to group countries according to specific features. A hierarchical cluster algorithm was used and the average dissimilarity between two matrices x and y (treated as vectors) was measured by the Canberra distance

$$d(x,y) \quad = \quad \sum_{k=1}^{n} \frac{|x_k - y_k|}{|x_k| + |y_k|}.$$

This choice was made because the entries of contact matrices range over several orders of magnitude, and this distance, differently from L_1 and L_2 distances, is appropriate to measure average relative rather than absolute dissimilarities [13]. Results are discussed in the main text.

As a complement on clustering, we look at the network of contacts within households, schools and workplaces and evaluate the frequencies of the number of links of every individual averaging over the modeled European countries (see Figure S14). We can observe that about 60% of the population has more than 20 contacts; more than 10% has maximum one contact, and the remaining frequencies are all below 6%, the lowest being 5 contacts.

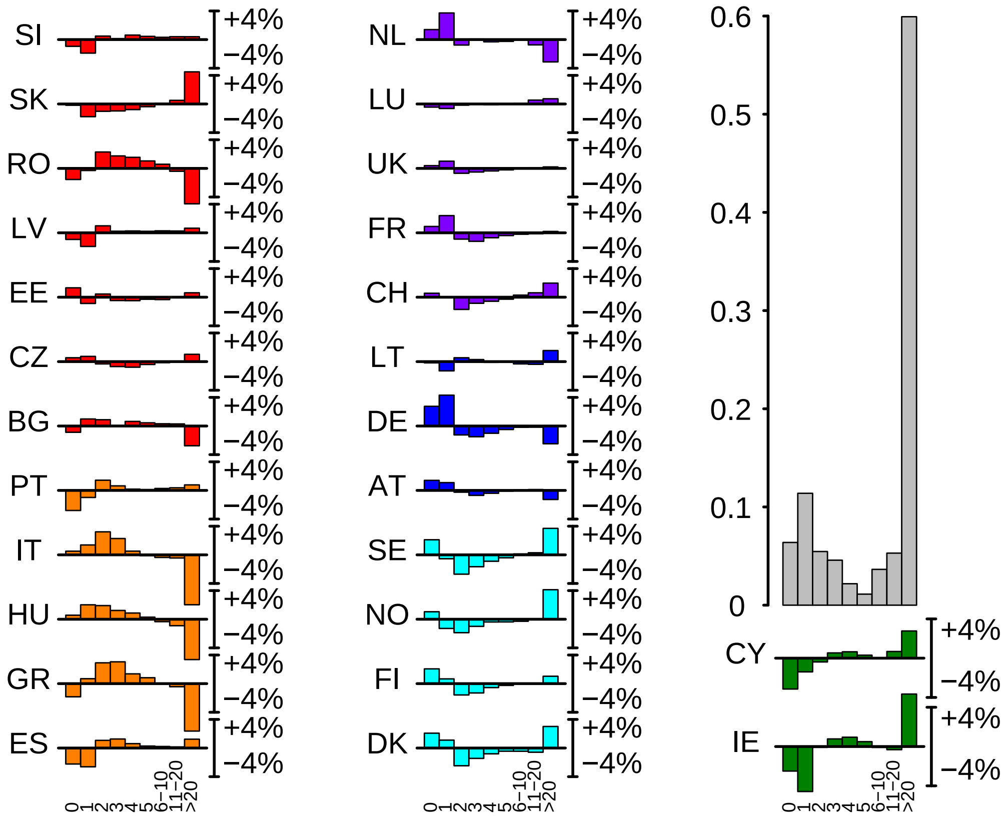

Figure S14: Grey bars (top right) represent the fractions of individuals having from zero to more than 20 contacts with household members and schoolmates/work colleagues, as obtained by averaging over the simulated European countries. Colored bars represent the deviations (in percentage) from the European average for every country. The colors of the bars identify countries belonging to the same cluster, as resulting from our clustering analysis (see main text).

Deviations from these values in the single countries highlight some well-defined features: for instance, Cyprus and Ireland have lower frequencies of individuals with zero or one contacts, slightly above the European values for the intermediate cases, and much higher frequencies of individuals with more than 20 contacts. This pattern can be explained in terms of the socio-demographic structure of these two countries: households tend to be large and populations are young, therefore people are likely to have a higher number of contacts. On the other hand, Italy, Hungary and Greece are characterized by high frequencies of intermediate number of contacts and very few individuals with more than 20 contacts, reflecting the fact that household size is above the European average but the fraction of people in schools (contributing to increase the average number of contacts) is much lower than in Cyprus and Ireland. Scandinavian countries, where households are smaller, all have higher frequencies of zero and more than 20 contacts, and lower frequencies of intermediate number of contacts.

3 Comparison with Polymod

We compare our contact matrices with those derived by the Polymod survey [14]. Since Polymod matrices contain the average number of contacts between individuals of different age classes,

while ours represent frequencies of adequate contacts, we multiply the total matrix for UK, Italy, Germany, Finland, Luxembourg and the Netherlands, as derived by our virtual society, by the sum of the elements of the corresponding Polymod matrix. (We remind that the sum of all elements of every single simulated total matrix is one). Moreover, we need to consider the same age classes as in Polymod, therefore we group individuals by 5-year age brackets, from 0–4 to 65–69, and the elderly into a unique class for the over 70.

For a visual comparison, we consider for each country the number of contacts that an individual belonging to a certain age group has with every age group (Figures S15, S16 and S17). We can observe that our results are in good agreement with Polymod: young individuals tend to have mostly same-age contacts; patterns in older age classes are smoother for our matrices, but they are always comprised in the values of Polymod matrices.

Other comparisons between our matrices and the Polymod ones are reported in the main text.

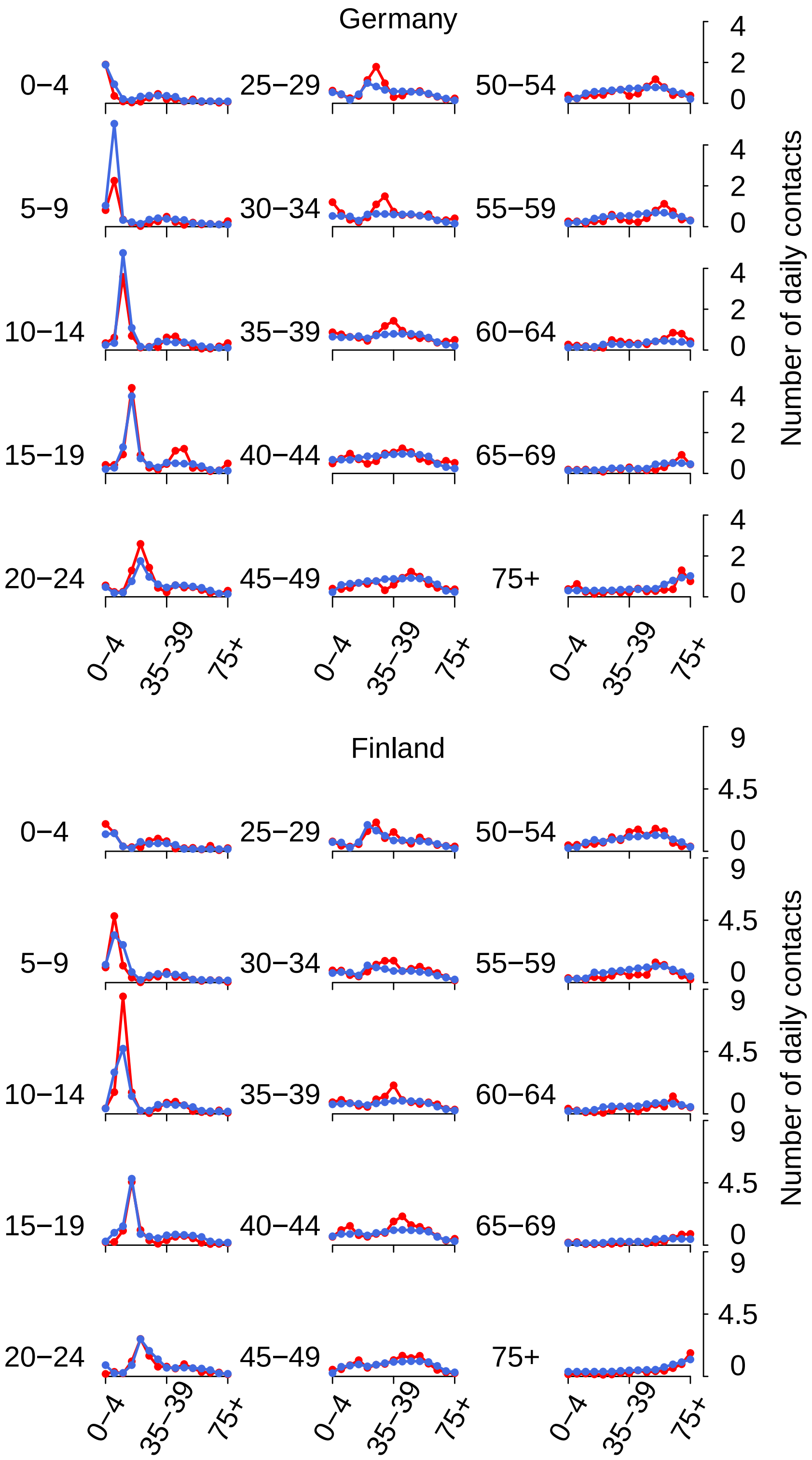

Figure S15: Average number of contacts that an individual of a 5-year class (except for individuals aged more than 75 who are grouped into a unique class) has with individuals of every age group in Germany (top) and Finland (bottom). Red line as obtained from the Polymod survey, blue line as resulting from our virtual society.

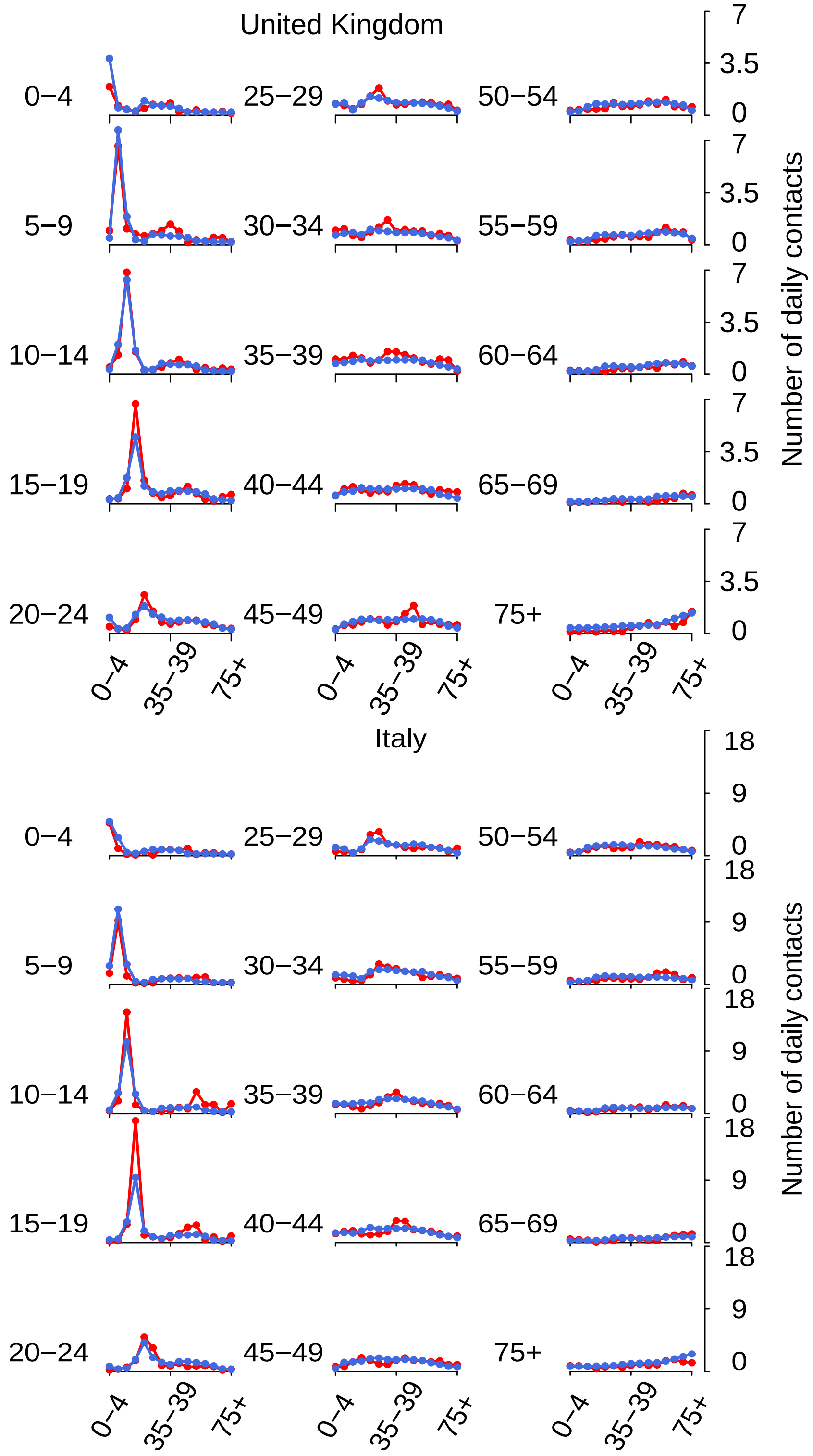

Figure S16: Average number of contacts that an individual of a 5-year class (except for individuals aged more than 75 who are grouped into a unique class) has with individuals of every age group in the United Kingdom (top) and Italy (bottom). Red line as obtained from the Polymod survey, blue line as resulting from our virtual society.

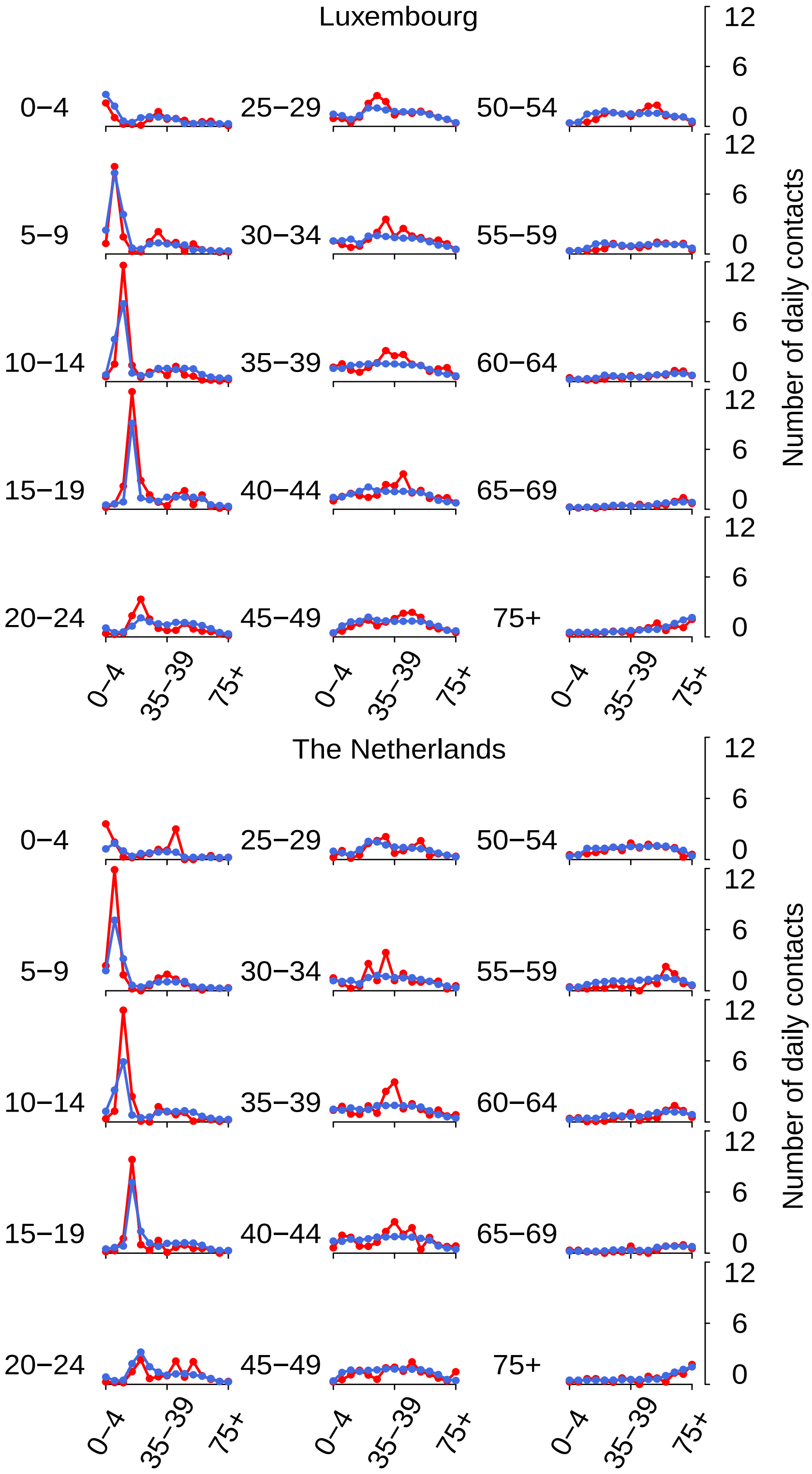

Figure S17: Average number of contacts that an individual of a 5-year class (except for individuals aged more than 75 who are grouped into a unique class) has with individuals of every age group in Luxembourg (top) and the Netherlands (bottom). Red line as obtained from the Polymod survey, blue line as resulting from our virtual society.

4 Simulation of a pandemic event

We consider a disease emerging in a completely susceptible population (e.g., as the case of an influenza pandemic); we assume $R_0 = 1.4$ and simulate an ordinary differential equation SIR (susceptible-infective-recovered) model, employing our contact matrices. The outcomes of the simulations based on our mixing patterns, presented in Figure S18, show a high prevalence among school-age children, becoming intermediate for working ages and progressively declining in the elderly; prevalence among little children is at an intermediate level. This pattern is mainly driven by country-specific employment and schooling rates. The shapes are similar for all countries; however, some differences are visible. For instance, the age at which prevalence starts declining is variable across countries, generally higher (around 60) in Northern Europe and lower (around 50) elsewhere and more markedly in Southern countries: this is probably an effect of the differences in retirement age across Europe. Moreover, the relative difference in prevalence between children and adults is variable across Europe.

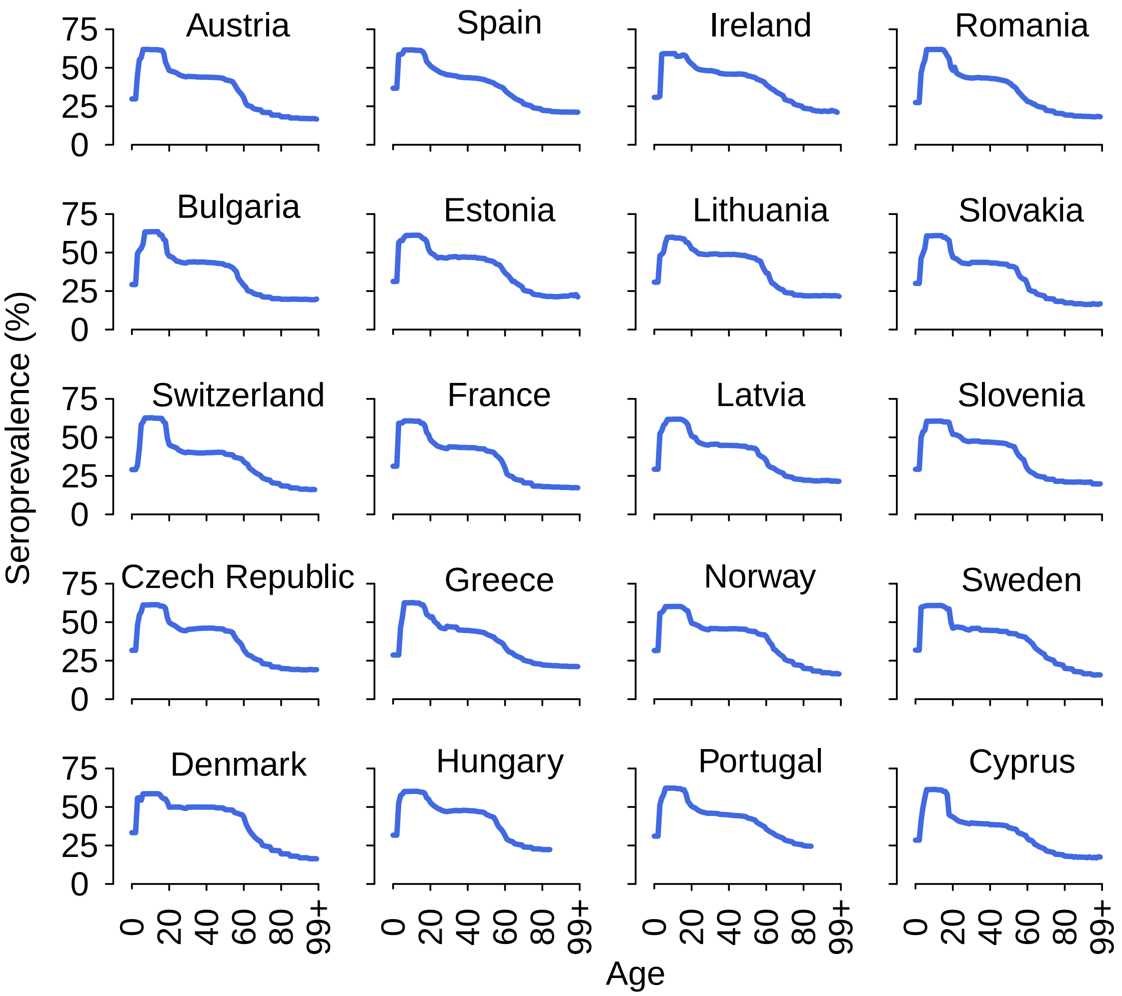

Figure S18: Seroprevalence profiles (percentage) for the countries not shown in the main text as obtained by simulating an SIR model with $R_0 = 1.4$, using our contact matrices and initializing the system with a fully susceptible population but for one infective individual aged 0.

5 Socio-demographic structure and disease epidemiology

We simulate a pandemic event using our contact matrices grouped by 5-year age brackets up to the class 70+; we assume a single value for the scale factor (namely, the one leading to $R_0 = 1.4$ in the UK). The most significant correlations between epidemiologically relevant quantities and socio-demographic characteristics are described in the main text. Other factors have been tested; we found significant correlations between the basic reproduction number R_0 and average age (Pearson correlation test -0.61, p-value 0.001), average household size (0.41, p-value 0.04), fraction of students aged less than 17 years (0.43, p-value 0.03), fraction of students attending primary school averaged over the duration of the school cycle (0.41, p-value 0.04). On the contrary, no significant correlation between R_0 and the fraction of inactive individuals was found.

It is expected that the basic reproduction number can be related to matrix assortativeness. We use the Q index [15], which is defined as

$$Q = \frac{\mathrm{Tr}(P) - 1}{n - 1}$$

where n is the number of age groups and P is the matrix whose elements p_{ij} represent the fraction of contacts that age group i has with age group j: $p_{ij} = M_{ij} / \sum_j M_{ij}$, where M is the contact matrix [15], as a measure of this characteristic.

The Q index results to be positively correlated to R_0 (0.41, p-value 0.04); this quantity however can not be derived *a priori* from census data, since it is a characteristic of contact matrices. Nonetheless, assortativeness is heavily related to the duration of primary school cycle (correlation -0.72, p-value < 0.001), therefore we consider it as a proxy for matrix assortativeness.

Notably, the duration of primary schools is not significantly correlated to R_0. However, the only factor among those previously mentioned that, added to average age in a two-variables multiregression model for R_0, gives a significant improvement with respect to the one with average age as the only independent variable, is the duration of primary school cycle.

As regards the attack rate, we found even more significant correlations with average age

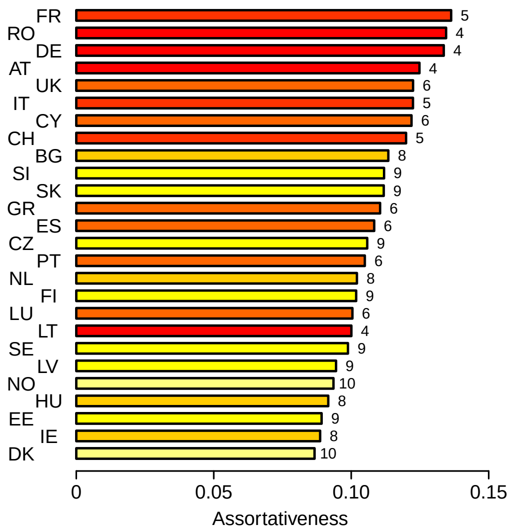

Figure S19: Assortativeness of the simulated contact matrices (population grouped by 5-year age brackets up to the class 70+). The numbers on the right of the bars represent the duration (in years) of the primary school cycle; colors from red to yellow are proportional to those numbers.

(-0.91, p-value< 0.001), average household size (Pearson correlation test 0.47, p-value 0.01), fraction of students aged less than 17 years (0.69, p-value < 0.001), fraction of students attending primary school averaged over the duration of the school cycle (0.76, p-value < 0.001). All the other factors, included matrix assortativeness, are not significantly correlated to the attack rate.

References

[1] Statistical Office of the European Commission (Eurostat) (2011). Database by themes. (Available at http://epp.eurostat.ec.europa.eu/portal/page/portal/statistics/search_database).

[2] Education, Audiovisual and Culture Executive Agency, European Commission. Key Data on Education in Europe 2009. (Available at http://eacea.ec.europa.eu/education/eurydice/key_data_en.php).

[3] Schweizer Medieninstitut für Bildung und Kultur. The Swiss Education System. (Available at http://www.educa.ch/en).

[4] UNESCO Institute for Statistics. International Standard Classification of Education - 1997 version. (Available at http://www.uis.unesco.org/Pages/default.aspx).

[5] Education, Audiovisual and Culture Executive Agency, European Commission. Key Data on Education in Europe 2005. (Available at http://eacea.ec.europa.eu/education/eurydice/key_data_en.php).

[6] Education, Audiovisual and Culture Executive Agency, European Commission (2010). National Education Systems and Policies. (Available at http://eacea.ec.europa.eu/education/eurydice/eurybase_en.php).

[7] National Statistics Institute, Bulgaria. Education and Lifelong Learning. (Available at http://www.nsi.bg/otrasalen.php?otr=36).

[8] Swiss Federal Statistical Office. Système d'éducation. (Available in French at http://www.bfs.admin.ch/bfs/portal/fr/index/themen/15/02/data/blank/01.html).

[9] Institut National de la Statistique ed des études économiques, France. Thémes: Enseignement-Éducation. (Available in French at http://www.insee.fr/fr/themes/theme.asp?theme=7).

[10] Department for Education, United Kingdom. Education and Training Statistics for the United Kingdom. (Available at http://www.education.gov.uk/rsgateway/DB/VOL/v000891/index.shtml).

[11] Department of Education and Skills, Central Statistics Office, Ireland. Early Start Preschool Programmes by County, Year and Statistic. (Available at http://www.cso.ie/en/index.html).

[12] Merler S, Ajelli M (2010) The role of population heterogeneity and human mobility in the spread of pandemic influenza. Proc R Soc B 277: 557.

[13] Critchlow D (1985) Metric Methods for Analyzing Partially Ranked Data, volume 34. Springer.

[14] Mossong J, Hens N, Jit M, Beutels P, Auranen K, et al. (2008) Social contacts and mixing patterns relevant to the spread of infectious diseases. PLoS Med 5: e74.

[15] Iozzi F, Trusiano F, Chinazzi M, Billari FC, Zagheni E, et al. (2010) Little-Italy: An Agent-Based Approach to the Estimation of Contact Patterns- Fitting Predicted Matrices to Serological Data. PLoS Comput Biol 6: e1001021.
